# A Novel Cluster-Based Approach for PPG Signal Preprocessing: Motion Artifact Filtering and Ambient Light Correction

**DOI:** 10.3390/s26206636

**Published:** 2026-10-20

**Authors:** Matteo Ricci, Daniel Suárez, Vicente Bertomeu-González, Arturo Martínez-Rodrigo, Raúl Alcaraz, José J. Rieta

**Affiliations:** 1 BioMIT.org, Electronic Engineering Department, Universitat Politècnica de València, 46022 Valencia, Spain; matricci@upv.es; 2 Benejúzar Health Center, Orihuela Health Department, 03390 Benejúzar, Spain; suarez_dan@gva.es; 3 Department of Clinical Medicine, Miguel Hernández University, 03202 Elche, Spain; vbertomeu@umh.es; 4 Research Group in Electronic, Biomedical and Telecommunication Engineering, University of Castilla-La Mancha, 16071 Cuenca, Spain; arturo.martinez@uclm.es (A.M.-R.); raul.alcaraz@uclm.es (R.A.)

**Keywords:** photoplethysmography, wearable sensors, motion artifacts, ambient light correction, accelerometer-based clustering, wavelet denoising, contact quality index, signal preprocessing

## Abstract

Photoplethysmography (PPG) is widely used in wearable devices, but wrist- and arm-worn recordings are sensitive to motion artifacts, ambient light, and changes in sensor–skin contact. This study presents an adaptive preprocessing pipeline that combines accelerometer-derived motion-state clustering, an ambient-light-based Contact Quality Index (CQI), and cluster-based Symlet 4 wavelet filtering. Motion states are obtained from less posture-dependent features of 10 s accelerometer windows, whereas the CQI is a heuristic indicator of ambient-light-related optical reliability, scaled from 0 to 1, that flags low-reliability segments without changing their motion label. The wavelet stage removes baseline drift, soft-thresholds the high-frequency detail bands with thresholds selected on training data, and preserves the bands that overlap the cardiac frequency range. The model was trained on 8 participants and evaluated on 27 held-out participants against a simultaneously recorded ECG. Compared with a band-pass filter and an accelerometer-referenced adaptive filter, the proposed method detected more beats (median sensitivity 1.00 vs. 0.91 and 0.92) and reduced the inter-beat-interval (IBI) error when missed beats were included (128.0 vs. 181.2 and 156.8 ms), particularly during movement, at the cost of a lower positive predictive value (0.92 vs. 1.00), whereas the reference filters yielded lower IBI error on detected beats (24.6 and 26.6 vs. 38.7 ms). The method also outperformed a fixed Daubechies 4 wavelet filter on all timing metrics. In a band-energy analysis, it reduced out-of-band energy by a median of 27.7 dB but also reduced useful-band energy by 41.0%. These results show that a lightly thresholded wavelet filter recovers more beats than fixed filters during daily-life movement, whereas the benefit of cluster-specific thresholds remains to be established in other cohorts and during higher-intensity activities.

## 1. Introduction

Photoplethysmography (PPG) is a non-invasive and cost-effective optical technique used in wearable devices for continuous health monitoring [1]. PPG enables the estimation of physiological variables such as heart rate (HR), oxygen saturation (SpO_2_), and, in some applications, blood-pressure-related indices [2]. The signal is generated by variations in light absorption associated with changes in blood volume in the microvascular bed of tissue. It contains a pulsatile AC component linked to cardiac cycles and a slowly varying DC component affected by respiration, thermoregulation, autonomic activity, tissue properties, and sensor coupling [3,4]. Because PPG devices are inexpensive, portable, and easy to deploy, they are increasingly used in primary-care, community, and free-living monitoring scenarios [5,6].

Despite these advantages, wearable PPG recordings are often degraded by disturbances that are not present, or are less severe, in controlled laboratory recordings. Motion artifacts, variable contact pressure, changes in sensor placement, and ambient light leakage can distort the waveform and reduce the reliability of downstream physiological analysis [7,8]. Conventional preprocessing methods, including fixed band-pass filters, smoothing, and generic noise-reduction algorithms, can reduce part of this contamination [9,10]. However, fixed filters are not always well suited to free-living data because artifact characteristics vary over time and may overlap the physiological frequency range [11,12]. Recent work has therefore moved toward artifact-aware quality assessment, reference-signal-less artifact detection, and computationally efficient wearable implementations rather than relying only on global filtering rules [13,14,15,16]. Contextual information from auxiliary sensors, especially accelerometers and optical ambient-light channels, can be useful for adapting the preprocessing strategy to the recording conditions [17,18].

Several recent studies are particularly relevant to this motivation. Wrist-PPG quality assessment in daily-life data has shown that segments suitable for heart-rate estimation are not necessarily suitable for morphology analysis, highlighting the need to report both artifact suppression and waveform preservation [13]. Patch-based and wearable heart-rate pipelines have also incorporated pre-quality checking, decomposition, and motion-aware reconstruction to improve robustness during movement [19,20,21]. In parallel, on-device signal-quality methods and lightweight neural architectures emphasize that preprocessing must remain computationally feasible for wearable deployment [14,16]. These developments support the use of explicit quality flags and context-dependent filtering rules in addition to conventional denoising.

This work proposes an adaptive PPG preprocessing pipeline that uses accelerometer and ambient-light information as contextual inputs. In this context, ambient-light correction does not refer to subtracting the ambient-light signal from the PPG, but to identifying the windows in which ambient light compromises the reliability of the optical signal and handling them with reliability-specific filtering parameters. The accelerometer signal is used to assign each 10 s window to a motion state. The ambient-light channel is used to compute a Contact Quality Index (CQI), a heuristic indicator of the reliability of the optical interface that flags segments affected by possible poor contact, detachment, or excessive light penetration. The CQI is not interpreted as another motion cluster; it is a quality covariate that selects reliability-specific filtering thresholds and flags low-reliability segments. The resulting PPG filter combines motion-state information, contact reliability, and Symlet 4 wavelet decomposition to attenuate non-physiological components while preserving the frequency bands expected to contain pulsatile PPG activity.

The main contributions of the study are: (i) a fully specified clustering procedure for accelerometer-derived motion states, with all model parameters reported; (ii) a formally defined CQI with a consistent direction, explicit normalization, and special handling of flat, saturated, or disconnected ambient-light segments; (iii) a quality-aware filtering strategy that separates motion labels from contact-quality flags; (iv) an energy and SNR analysis with units tied to the PSD integration procedure; and (v) a beat-level validation against a simultaneously recorded ECG in participants not used for model fitting. The method is evaluated on long-duration recordings acquired under daily-life conditions, and its performance is compared with fixed band-pass and fixed wavelet reference filters and with an accelerometer-referenced adaptive filter, both in representative examples and at the beat level. The claims are intentionally limited to the evaluated recordings because broader generalization requires independent validation across additional devices, placements, and subject populations.

## 2. Materials and Methods

### 2.1. Data Collection

The dataset consists of physiological recordings acquired with the Polar Verity Sense device (Polar Electro Oy, Professorintie 5, 90440 Kempele, Finland). The device provides three PPG channels, one ambient-light channel, and an integrated tri-axial accelerometer. Its optical sensor comprises six green LEDs arranged in a ring around a single central photodetector (https://github.com/polarofficial/polar-ble-sdk/issues/202, accessed on 14 October 2026); green light is commonly used in wrist- and arm-worn heart-rate sensors because its shallow penetration into the tissue makes the signal less sensitive to motion than red or infrared light, although it does not eliminate motion artifacts [3,4]. Through the official Polar software  development kit (SDK, version 5.2.0), the device streams the optical signal as four channels with 22-bit resolution, namely three PPG channels and one ambient-light channel, at selectable sampling rates; in this study, the signals were streamed at 55 Hz to the acquisition application. The ambient-light channel is provided separately from the PPG channels, and the manufacturer does not document whether any additional ambient-light compensation is applied internally. In the present analysis, the ambient-light channel was not subtracted from the PPG channels and was used only to compute the CQI (Section 2.4.1). In addition, participants wore a Polar H10 chest strap (Polar Electro Oy), which records a single-lead ECG at 130 Hz and was paired with the same acquisition application; this ECG, whose RR intervals have been validated against a Holter ECG [22], was used only as an independent reference for the beat-level validation described in Section 2.6. For the purposes of this study, the PPG signal analyzed was obtained as the sample-wise average of the three raw PPG channels provided by the device, yielding a single composite waveform used throughout the preprocessing and evaluation pipeline. This choice was based on a preliminary analysis of recordings from 10 participants of different sex, age, and physical characteristics. The delay between channels, estimated segment by segment by cross-correlation on artifact-free segments with channel 0 as reference, was always within ±0.5 samples (approximately ±9 ms at 55 Hz), which is negligible compared with the duration of a cardiac cycle. In addition, a weighted mean in which each channel was weighted segment by segment according to its signal-to-noise ratio produced a waveform that was visually almost identical to the arithmetic mean, while introducing small discontinuities at segment boundaries. The arithmetic mean was therefore adopted as a simple, parameter-free fusion rule, as also suggested by the developers of the official Polar software development kit (https://github.com/polarofficial/polar-ble-sdk/issues/202, accessed on 14 October 2026). Devices were distributed to 50 patients with heterogeneous clinical profiles, including cardiovascular conditions. Clinical data were available for 46 of them; their characteristics are summarized in Table 1, both for these 46 participants and for the 33 analyzed participants (Section 2.6) for whom clinical data were available. The participants with clinical data had a mean age of 54.2 years, 39% were women, the mean body mass index was 27.9 kg/m^2^, and 67% had hypertension, defined as a blood pressure of at least 140/90 mmHg or treatment with antihypertensive medication [23].

**Figure 1:**
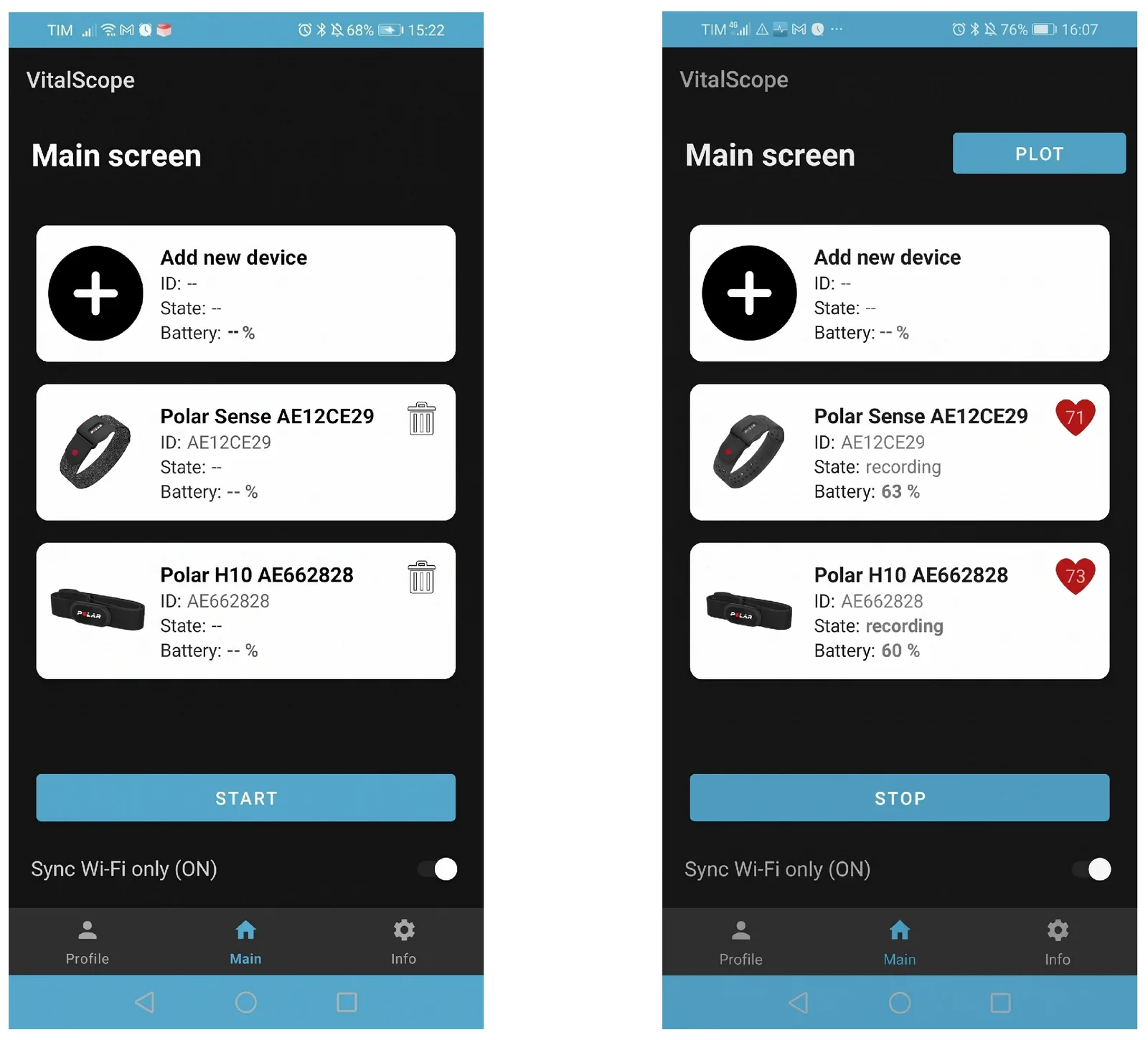
Vital Scope application used during data acquisition. The left panel shows the main screen after pairing the Polar Verity Sense and the Polar H10 chest strap, before the start of the recording; because the devices are not yet connected, their state and battery level are not available (“–”). The right panel shows the same screen during recording, with the battery level and the heart rate currently measured by each device. The first card (“Add new device”) is the button used to pair a new sensor, and its fields are placeholders.

This dataset was selected because it contains synchronized optical and inertial signals recorded with the same wearable platform used in the proposed preprocessing pipeline. Each participant received the device and written instructions on placement, charging, and recording. The instructions covered continuous 24 h acquisition using the Vital Scope application developed by the research team at the University of Castilla-La Mancha (Spain). Participants installed the application, wore the device on the forearm, paired the sensor, and initiated recording. Figure 1 shows the application screens used for device pairing and recording. The application uploaded the acquired data to a server, after which the files were imported to a local computer for analysis.

**Figure 2:**
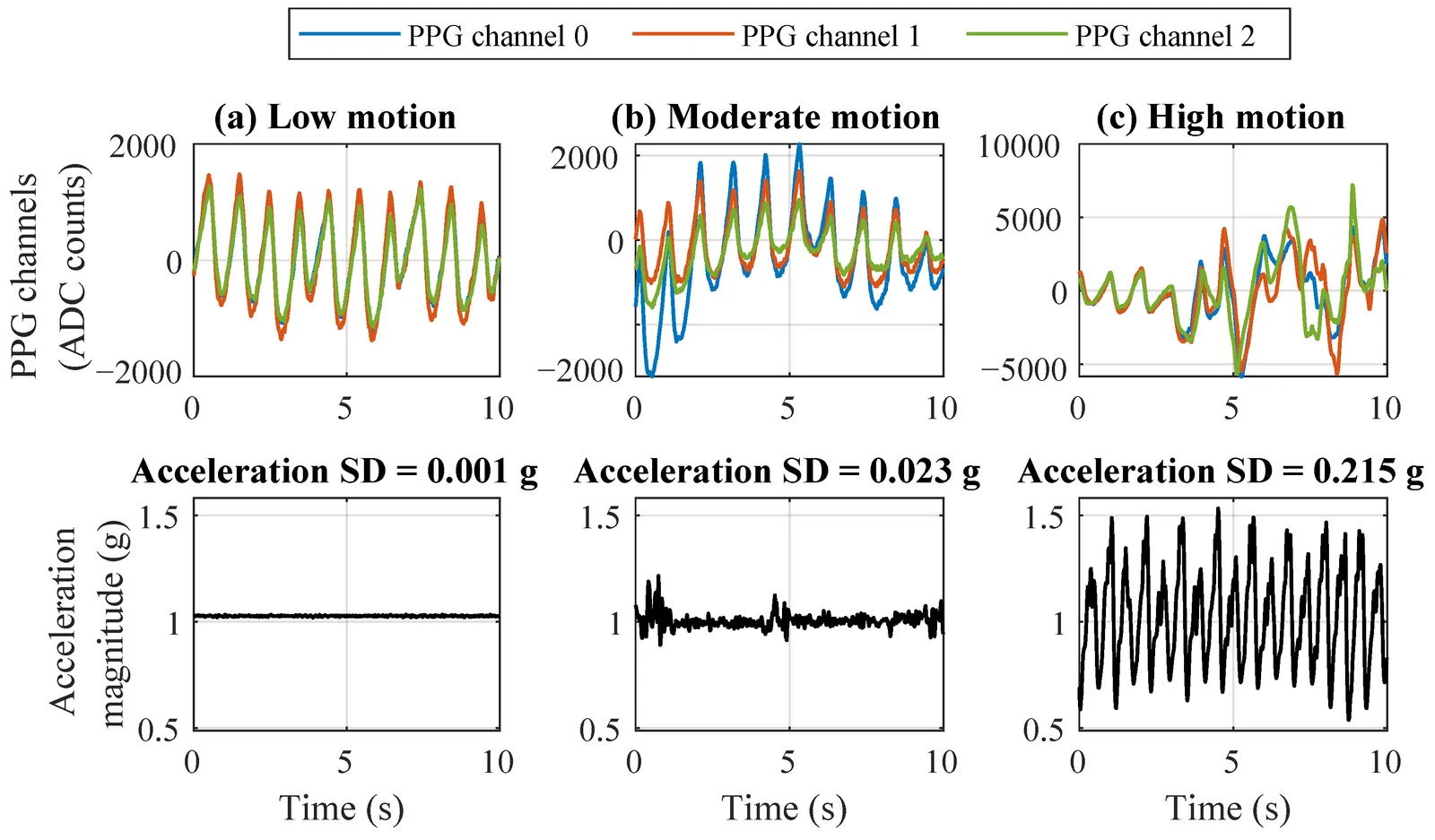
Raw PPG channels (top) and acceleration magnitude (bottom) in three test windows with (**a**) low, (**b**) moderate, and (**c**) high motion, selected at the 25th, 50th, and 95th percentiles of the acceleration standard deviation within clusters 1, 2, and 3, respectively. The slow baseline drift (2 s moving average) was removed from the PPG channels for display only; amplitudes are in raw ADC counts. The acceleration magnitude includes gravity and is shown on the same scale in all panels.

During the 24 h sessions, participants performed their usual daily activities, including resting, walking, and light tasks; no structured exercise, such as running, was included in the protocol, and the activities were not annotated. The PPG and ambient-light channels were acquired at 55 Hz, which was the sampling rate available for the optical data in this configuration. This rate is adequate for the heart-rate-oriented preprocessing and quality-assessment analyses performed here, but it should not be interpreted as sufficient for every possible cardiovascular endpoint. The accelerometer stream had a nominal raw sampling rate of 52 Hz in the exported files. Therefore, accelerometer-specific filtering was performed at 52 Hz, and the resulting acceleration signal was then resampled to the 55 Hz optical time base. After resampling, all 10 s windows were defined on the common 55 Hz grid so that each acceleration segment corresponded to the same time interval as the PPG and ambient-light segments. Continuous recording blocks were defined by splitting the signals at gaps longer than 1 s. Within each block, the PPG, ambient-light, and acceleration signals were linearly interpolated onto the common 55 Hz grid, short missing segments were filled by linear interpolation, and only complete 10 s windows were analyzed. Figure 2 shows the three raw PPG channels and the acceleration magnitude in three test windows with low, moderate, and high motion. At rest, the three channels show nearly identical pulsatile waveforms; during high motion, all three channels are distorted by motion artifacts and differ more from one another.

### 2.2. Preprocessing Pipeline

**Figure 3:**
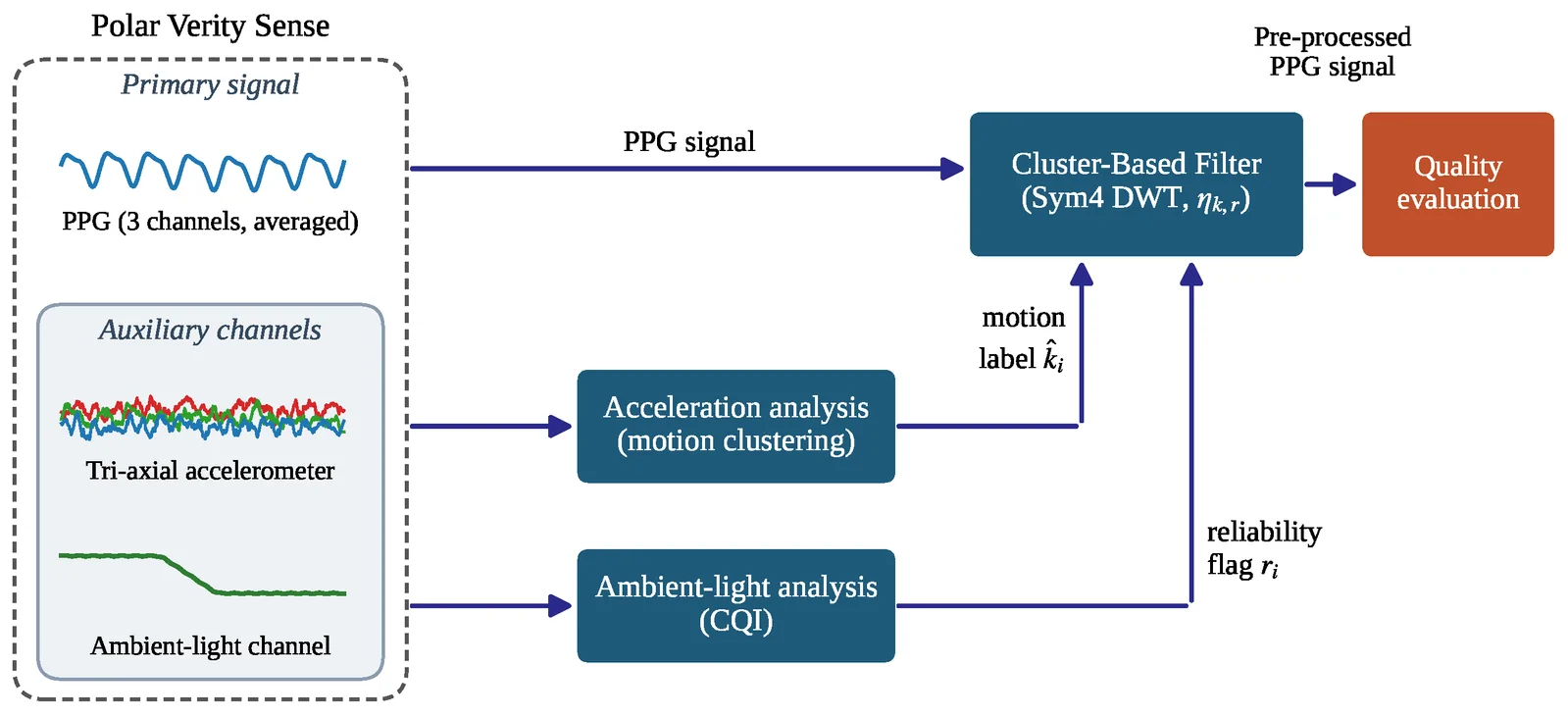
Overview of the proposed preprocessing pipeline. The PPG signal, obtained as the average of the three PPG channels, is the primary signal to be filtered. The tri-axial accelerometer and the ambient-light channel are auxiliary channels: the former provides the motion-state label ${\hat{k}}_{i}$ and the latter the reliability flag $r_{i}$ derived from the CQI. These contextual inputs select the filtering parameters but are not combined with the PPG waveform. The blue waveform represents the PPG signal, the three colored traces represent the three accelerometer axes, and the green trace represents the ambient-light signal; dark blue boxes indicate the processing stages of the proposed pipeline, the orange box indicates the downstream quality evaluation, and arrows indicate the signal flow.

The preprocessing pipeline uses all available signal sources from the device while preserving a clear role for each channel. The PPG signal is the primary signal to be filtered. The accelerometer signal provides a motion-state label, and the ambient-light signal provides an independent contact-quality flag. This separation is consistent with recent pipelines that first identify unreliable or motion-corrupted intervals and then select an appropriate correction or estimation rule, rather than applying the same operation to all segments [15,19,21]. As illustrated in Figure 3, auxiliary channels do not replace the PPG; instead, they define contextual rules that guide the filtering stage. The goal is to reduce motion-related and contact-related artifacts while preserving the morphology required for downstream signal-quality assessment and physiological analysis.

### 2.3. Accelerometer Data Analysis

Tri-axial accelerometer data were extracted from the raw files with their timestamps. The three channels, denoted as accX, accY, and accZ, were originally expressed in milligravity units (mg) and converted to gravitational units (g). A scalar acceleration magnitude was then computed as

$$A\left[n\right]=\sqrt{\mathrm{accX}{\left[n\right]}^{2}+\mathrm{accY}{\left[n\right]}^{2}+\mathrm{accZ}{\left[n\right]}^{2}}.$$

**Figure 4:**
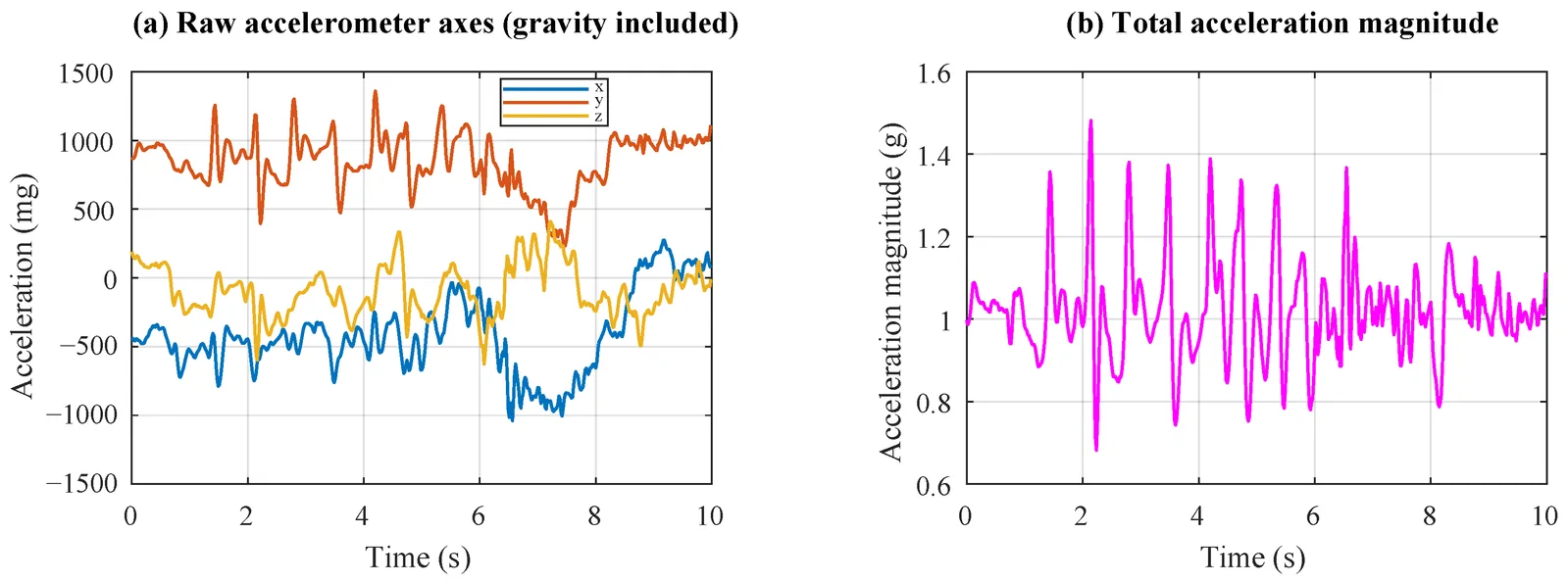
Representative 10 s accelerometer segment. Panel (**a**) shows the three raw acceleration axes, and panel (**b**) shows the total acceleration magnitude computed from the same samples. The magnitude summarizes the three axes but still includes both posture and dynamic motion components.

This magnitude contains both dynamic acceleration and the gravity component. Consequently, it should be interpreted as total acceleration, which reflects posture and motion jointly, not as pure movement intensity. Figure 4 illustrates the three acceleration axes and the corresponding total acceleration magnitude for a representative 10 s interval. To reduce the effect of posture on the clustering stage, the final clustering features were selected from statistics that emphasize within-window variation and distribution shape rather than the absolute mean level.

The total acceleration signal was low-pass filtered with a second-order Butterworth filter with a 5 Hz cutoff at the accelerometer sampling rate of 52 Hz. This step removes high-frequency sensor noise and abrupt disturbances that are not relevant for the motion-state analysis. The filtered acceleration signal was then resampled to the 55 Hz optical time base before segmentation. Signals from different files and participants were processed with the same pipeline and then combined for feature extraction and model evaluation.

#### Acceleration-Based Clustering

The synchronized acceleration magnitude was segmented into non-overlapping 10 s windows. This duration balances temporal resolution with stable statistical feature estimation and is consistent with common practice in activity-recognition tasks [24]. For each window, seven candidate features were computed from the total acceleration samples $x_{1},\ldots ,x_{N}$, as shown in Table 2. For entropy, the continuous values were first discretized into B=20 equally spaced bins over the range observed in the training data. If $p_{b}$ is the empirical probability of bin *b*, bins with $p_{b}=0$ were excluded from the logarithmic sum.

**Figure 5:**
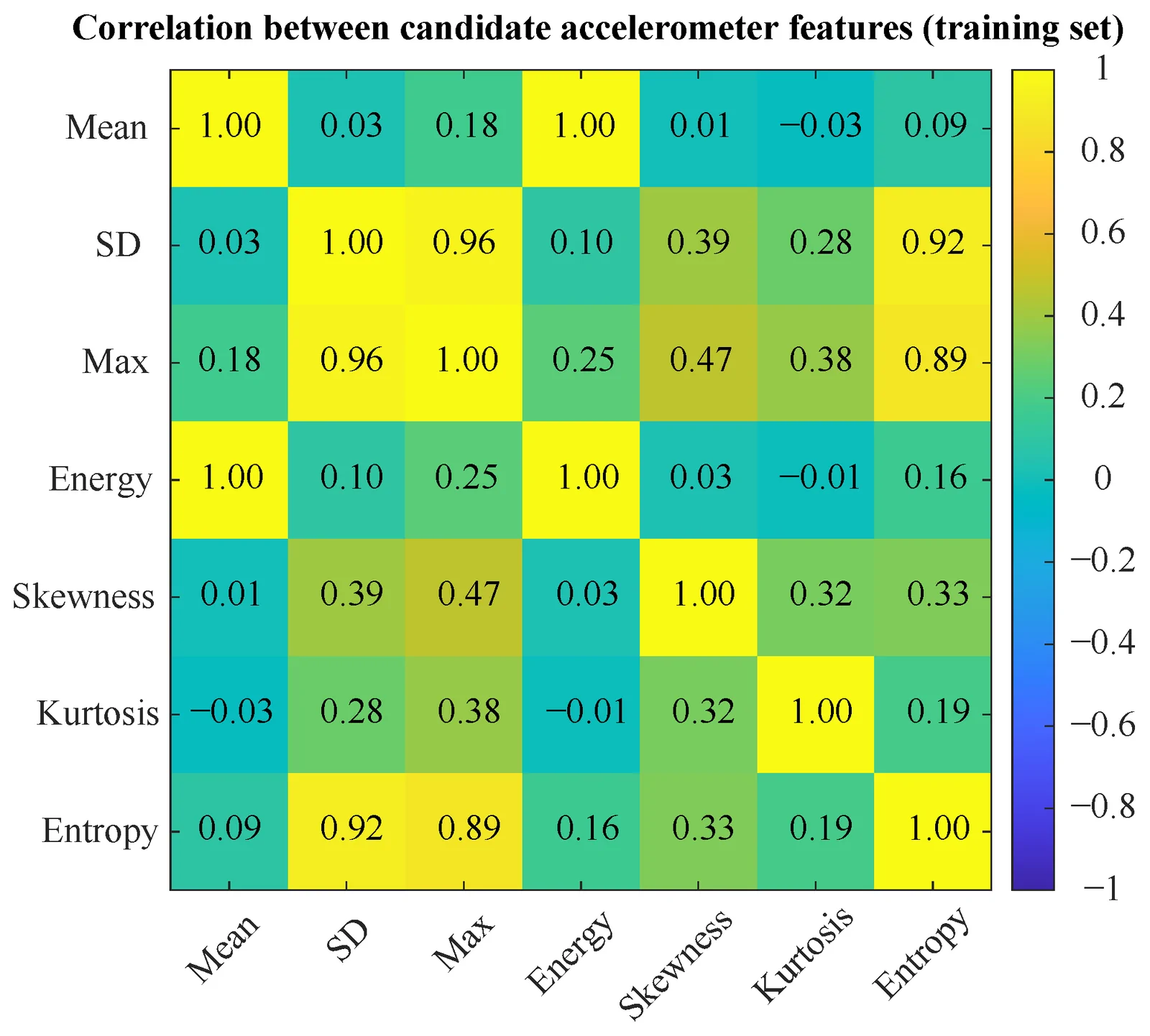
Correlation matrix of the normalized candidate accelerometer features. Values are Pearson correlation coefficients computed on the training windows; yellow tones indicate positive correlations, blue tones indicate negative correlations, and values close to zero indicate weak linear dependence between features.

Outliers in the candidate feature set were detected using the interquartile range (IQR) rule [25] and excluded when fitting the model. Min–Max normalization was then applied using only the training data parameters [26]. For a feature *z*, the normalized value was

$$\tilde{z}=\frac{z-z_{\min}^{\mathrm{train}}}{z_{\max}^{\mathrm{train}}-z_{\min}^{\mathrm{train}}},$$

with values clipped to [0,1] when applying the model to later sessions. The training minimum and maximum values are part of the model parameters and must be retained together with the final cluster centroids. Figure 5 shows the correlation structure among the normalized candidate features and was used to identify redundant feature pairs before clustering-based feature selection.

Feature selection combined correlation analysis and clustering quality. All possible pairs of candidate features were evaluated with k-means clustering. For each pair, k-means was run with 50 random initializations and a maximum of 1000 iterations, retaining the solution with the lowest within-cluster sum of squares. Candidate values k=2,3,4,5 were examined. Although the validity indices were slightly higher for k=2 (Silhouette 0.71 vs. 0.65 for k=3), k=3 was selected because it provided an interpretable separation between rest/low-motion, moderate-motion, and dynamic-motion states without creating small unstable clusters. Cluster quality was assessed using the Silhouette score [27], the Davies–Bouldin index [28], and the Dunn index [29], as defined in Table 3.

Before combining the indices, each metric was normalized across the candidate feature pairs. Let ${\bar{S}}_{\mathrm{silhouette}}$, ${\bar{S}}_{\mathrm{DBI}}$, and ${\bar{S}}_{\mathrm{Dunn}}$ denote the normalized scores, with the DBI reversed so that larger values are better. Let $P_{\mathrm{corr}}=\left|\rho \right|$ be the absolute Pearson correlation between the two features. The combined score was

$$S_{\mathrm{pair}}=0.25{\bar{S}}_{\mathrm{silhouette}}+0.25{\bar{S}}_{\mathrm{DBI}}+0.25{\bar{S}}_{\mathrm{Dunn}}-0.25P_{\mathrm{corr}}.$$

**Figure 6:**
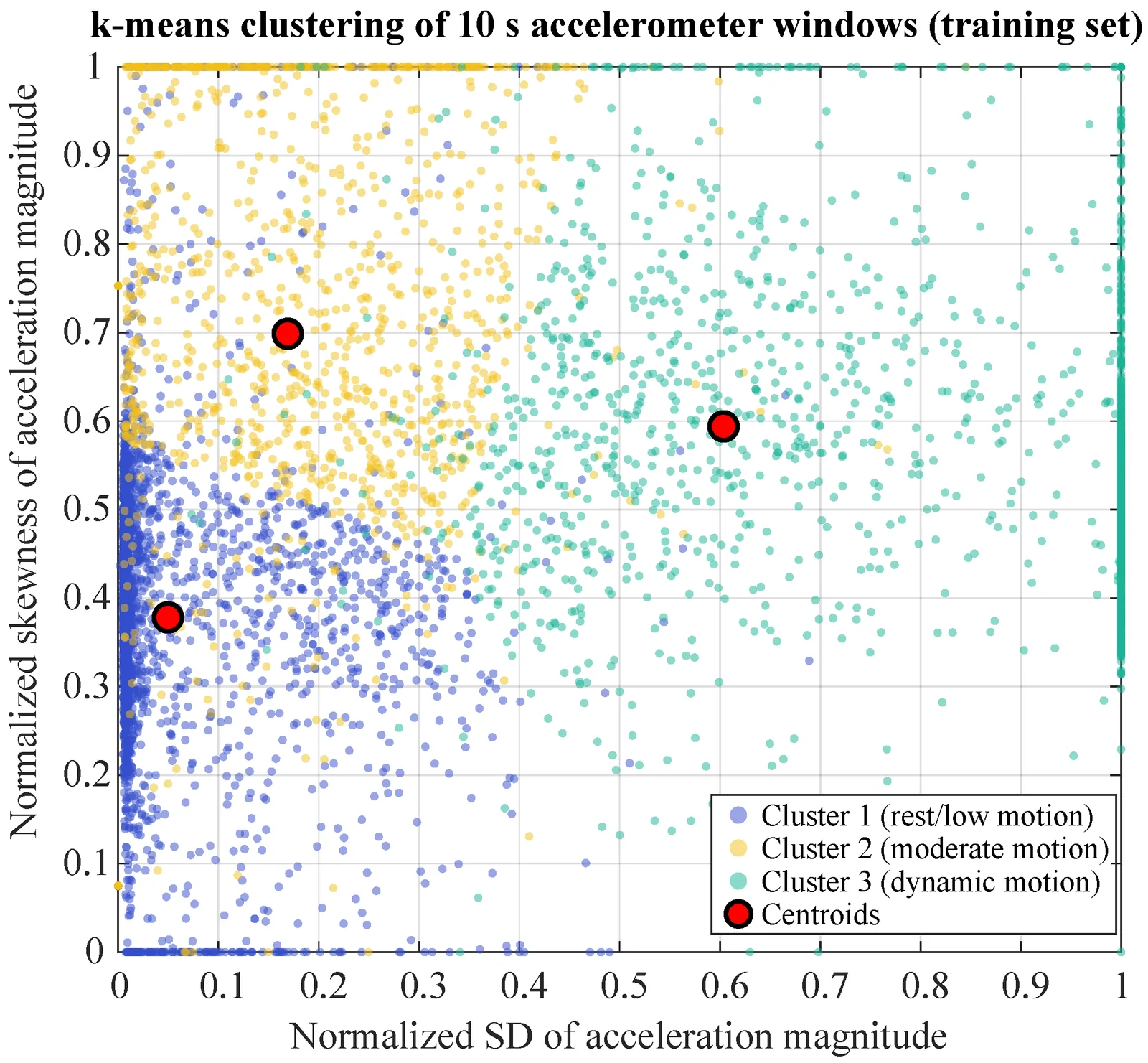
K-means clustering of 10 s accelerometer windows using normalized standard deviation and skewness. Each point represents one training window, colors indicate the three motion-state clusters, and red markers indicate the learned centroids in the normalized feature space (Section 2.6).

On the training windows, the highest combined scores were obtained by pairs that included a level-dependent feature and were strongly correlated, such as maximum intensity and entropy (|ρ|= 0.89) or standard deviation and entropy (|ρ|=0.92); the correlation penalty was therefore not sufficient to exclude redundant pairs. Because the absolute level of the total acceleration magnitude includes the gravity component and thus depends on posture, standard deviation and skewness were retained as clustering features on physiological grounds rather than on the combined score alone. These two features describe within-window movement variability and distribution shape, are weakly correlated (|ρ|= 0.39), and are less affected by static posture than level-dependent features such as the mean, maximum, energy, or entropy. The k-means results obtained with these two features are shown in Figure 6. The learned centroids $c_{1},c_{2},c_{3}$ in normalized standard-deviation–skewness space, together with the training Min–Max parameters, define the classifier used for subsequent sessions; they are not re-estimated during testing. Their numerical values are reported in Section 2.6.

Each 10 s PPG segment was assigned the motion label of the temporally corresponding accelerometer segment. The resulting labels describe motion state only: cluster 1 corresponds to rest/low-motion windows, cluster 2 to moderate-motion windows, and cluster 3 to dynamic-motion windows. These labels are relative to the movement intensities observed in this daily-life dataset, which consisted mainly of low-intensity activities: dynamic motion denotes the highest movement levels present in these recordings rather than vigorous exercise, and the clusters were not validated against annotated activities. Contact quality and ambient-light contamination are handled separately through the CQI described below.

### 2.4. Ambient Light Channel Analysis

**Figure 7:**
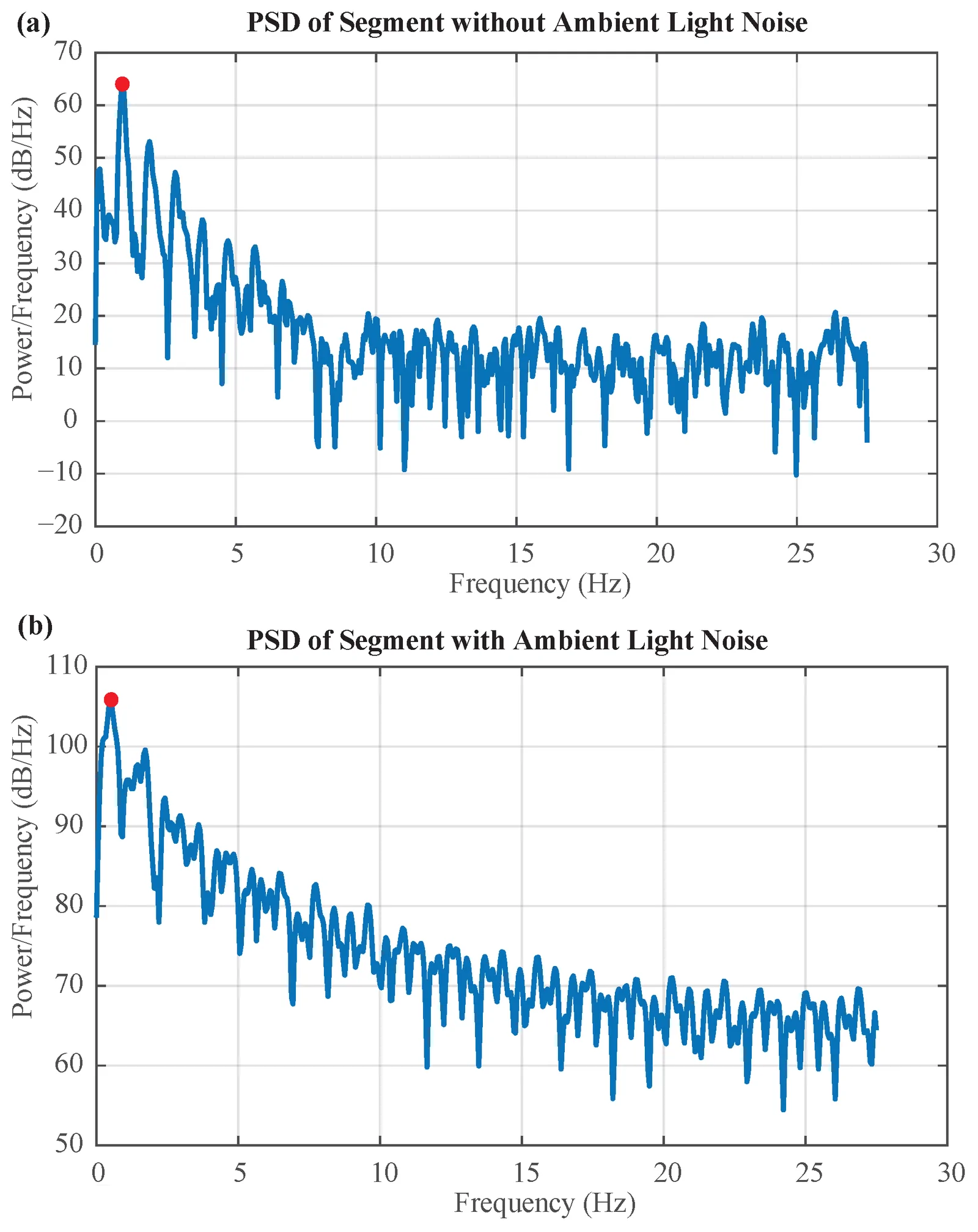
Representative ambient-light PSD patterns. Panel (**a**) shows a segment with stable sensor placement and low ambient-light spectral power. Panel (**b**) shows a segment affected by external light penetration, characterized by increased low-frequency power. In both panels, the blue line is the PSD estimated with Welch’s method, and the red dot marks its maximum.

The ambient-light channel was analyzed to detect external light penetration, poor sensor coupling, and possible sensor detachment. This channel is used as a practical proxy for optical coupling because recent studies have shown that sensor contact conditions, contact pressure, posture, and activity level can substantially change wrist or finger PPG quality, morphology, and heart-rate accuracy [30,31,32]. Power spectral density (PSD) estimation was used because changes in sensor contact can modify the distribution of ambient-light power across frequency [33]. As illustrated in Figure 7, poor positioning or partial detachment can increase low-frequency ambient-light power below approximately 2 Hz. Conversely, a nearly flat or zero-power ambient-light segment can indicate complete occlusion, saturation, or disconnection, depending on the raw amplitude and variance.

The ambient-light signal was divided into 10 s windows on the common 55 Hz time grid. For each segment, the mean was removed and the PSD was estimated using Welch’s method with 256-sample Hamming windows and 50% overlap. The MATLAB R2024a (The MathWorks, Inc., Natick, MA, USA) function pwelch was used with an FFT size of 2048 points. The area under the PSD curve was computed with the trapezoidal rule over frequency using trapz. Because the optical signal is stored in raw digital units, these PSD integrals are interpreted as relative spectral-energy measures and are compared through ratios or decibel changes rather than as absolute physical energy.

#### 2.4.1. CQI: Contact Quality Index

**Figure 8:**
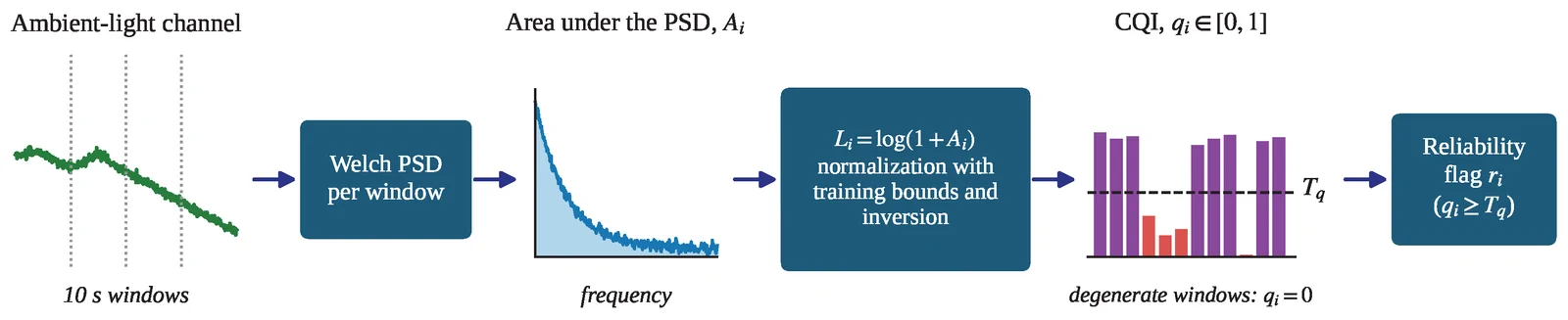
Workflow used to compute the Contact Quality Index (CQI). The ambient-light channel is segmented into 10 s windows, the PSD of each window is estimated with Welch’s method, and the area under the PSD, $A_{i}$, is computed. The logarithmic area $L_{i}=\log\left(1+A_{i}\right)$ is normalized with the bounds estimated on the training recordings and inverted to obtain the CQI, $q_{i}\in \left[0,1\right]$; degenerate windows are assigned $q_{i}=0$. Windows with $q_{i}\geq T_{q}$ are flagged as reliable ($r_{i}=1$, purple bars) and the others as low reliability ($r_{i}=0$, red bars). The green trace is the ambient-light signal, dotted vertical lines mark the window boundaries, the blue curve and shaded area show the PSD and its area $A_{i}$, the dashed line indicates the threshold $T_{q}$, dark blue boxes indicate the processing steps, and arrows indicate the processing flow.

The CQI computation workflow is summarized in Figure 8. The Contact Quality Index (CQI) is defined as a normalized reliability metric computed from the ambient-light PSD area. Its direction is fixed throughout the manuscript: $q_{i}=1$ denotes low ambient-light interference, that is, a reliable optical interface, whereas $q_{i}=0$ denotes possible poor contact, detachment, saturation, or excessive external light. For segment *i*, let $A_{i}$ be the PSD area of the ambient-light channel, and let

$$L_{i}=\log\left(1+A_{i}\right).$$

For non-degenerate segments, the CQI is computed as

$$q_{i}=1-\min\;\left(\max\;\left(\frac{L_{i}-L_{\min}}{L_{\max}-L_{\min}},\;0\right),\;1\right),$$

where $L_{\min}$ and $L_{\max}$ are the 1st and 99th percentiles of $L_{i}$ over the non-degenerate windows of the training recordings (Section 2.6). Because these bounds are fixed, the CQI is comparable across recordings and is not relative to the distribution of each individual recording. The inversion is used because larger ambient-light PSD area indicates stronger external light contamination or poorer coupling in the evaluated recordings. Degenerate cases are handled before normalization. A segment is marked unreliable and assigned $q_{i}=0$ if $A_{i}<\epsilon_{A}$, if the temporal variance of the ambient-light segment is below $\epsilon_{v}$, indicating a flat or disconnected signal, or if more than a fraction $r_{\mathrm{sat}}$ of its samples are equal to the segment maximum, indicating saturation or clipping. In this study, $\epsilon_{A}=0$, $\epsilon_{v}=1$ (squared raw digital units), and $r_{\mathrm{sat}}=0.5$.

**Figure 9:**
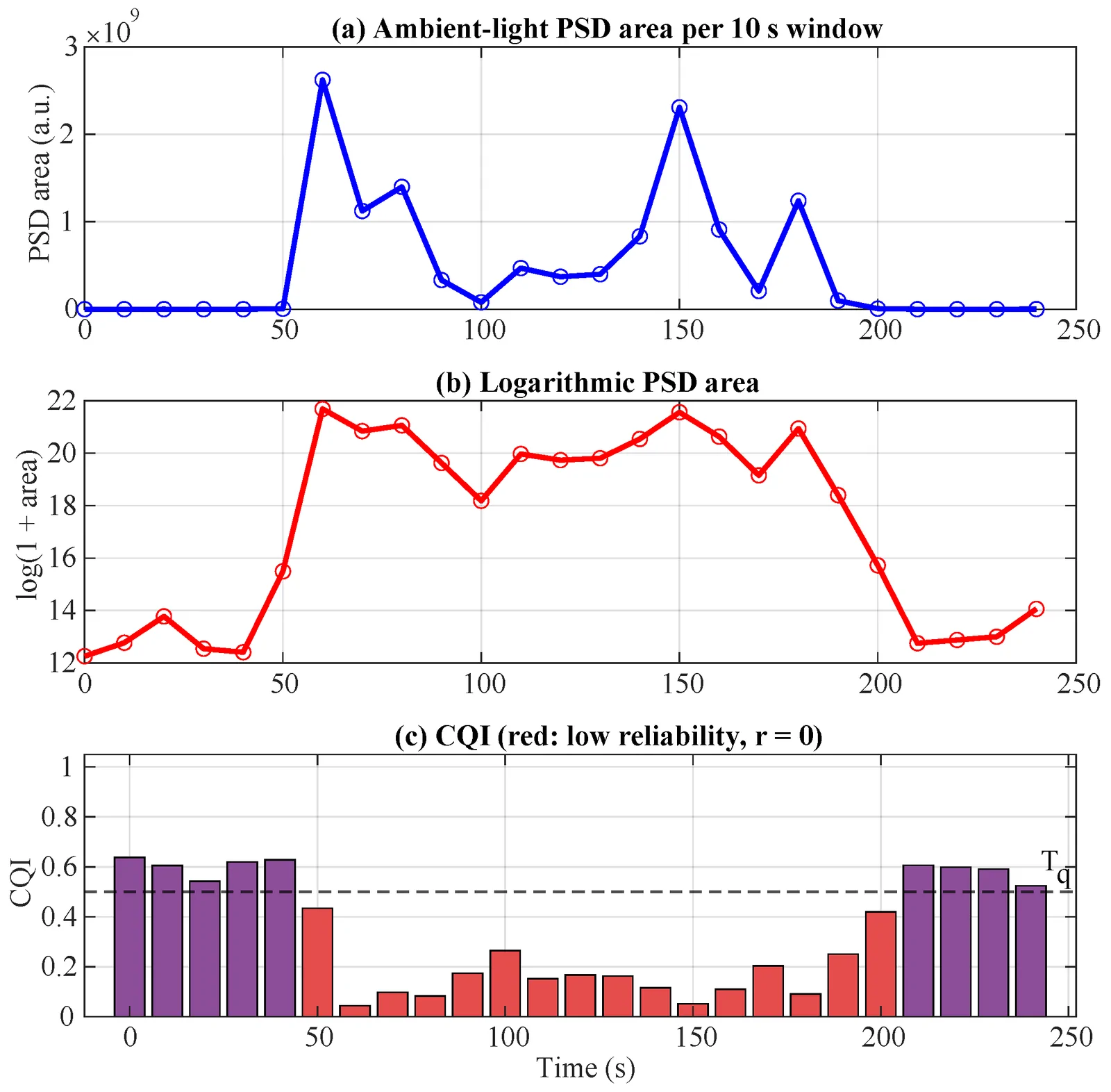
Example of CQI construction across consecutive segments. Panel (**a**) shows the ambient-light PSD area of 25 consecutive 10 s windows, panel (**b**) the logarithmic PSD area, and panel (**c**) the resulting CQI, with low-reliability windows ($q_{i}<T_{q}$) in red. The sequence was selected from the test recordings to illustrate a transient drop of the CQI below the threshold. Higher CQI values indicate lower ambient-light interference and, presumably, more reliable sensor–skin contact.

Figure 9 illustrates the effect of the logarithmic transform and normalization on a sequence of segments that includes a transient loss of reliability. Segments with low CQI values are marked as unreliable. The threshold used in this study was $T_{q}=0.5$, the midpoint of the scale; it was set a priori and was not optimized. This threshold is a preprocessing parameter and should be optimized or validated when the method is transferred to another device, placement, or recording protocol. Recent signal-quality studies support this conservative interpretation because the quality level required for simple heart-rate tracking can be lower than the quality required for morphology-sensitive analyses [13,14]. The CQI is a heuristic indicator derived only from the ambient-light channel. It has not been validated against independent measurements of sensor–skin contact, such as contact pressure or annotated detachment events, and it cannot distinguish reduced contact from ambient light that reaches the photodetector through the tissue while contact remains stable. It should therefore be interpreted as an indicator of ambient-light-related optical reliability rather than as a direct measure of contact quality. The name Contact Quality Index is retained because reduced sensor–skin contact is one of the main mechanisms by which ambient light reaches the photodetector, but the index does not measure contact itself.

#### 2.4.2. Reliability Flag and Motion-Label Smoothing

The CQI is incorporated as a quality flag rather than as a motion feature. For each segment *i*, the acceleration classifier first assigns a motion cluster

$${\hat{k}}_{i}=\arg\min_{k\in \{1,2,3\}}{∥{\tilde{x}}_{i}-c_{k}∥}_{2},$$

where ${\tilde{x}}_{i}$ is the normalized standard-deviation–skewness feature vector and $c_{k}$ is the centroid of motion cluster *k*. The CQI then assigns a reliability flag

$$r_{i}=\left\{\begin{array}{cc} 1, & q_{i}\geq T_{q},\\ 0, & q_{i}<T_{q}. \end{array}\right.$$

The final segment label is therefore the pair $\left({\hat{k}}_{i},r_{i}\right)$. The motion cluster ${\hat{k}}_{i}$ is not changed solely because contact quality is poor. Instead, low-reliability segments $\left(r_{i}=0\right)$ are filtered with their own threshold multipliers (Section 2.6) and can be flagged for downstream exclusion or manual review. This separation avoids assigning ambient-light artifacts to a motion cluster and keeps the interpretation of each label clear.

When short isolated labels occur, temporal smoothing is applied only to the motion labels and only among high-reliability segments. A label ${\hat{k}}_{i}$ is replaced by the majority label in a three-segment window {i−1,i,i+1} if $r_{i}=1$, both neighbors have r=1, and the two neighbors agree. Ties are left unchanged. Low-CQI segments are not used to force a motion-state change.

### 2.5. Cluster-Based Filtering

**Figure 10:**
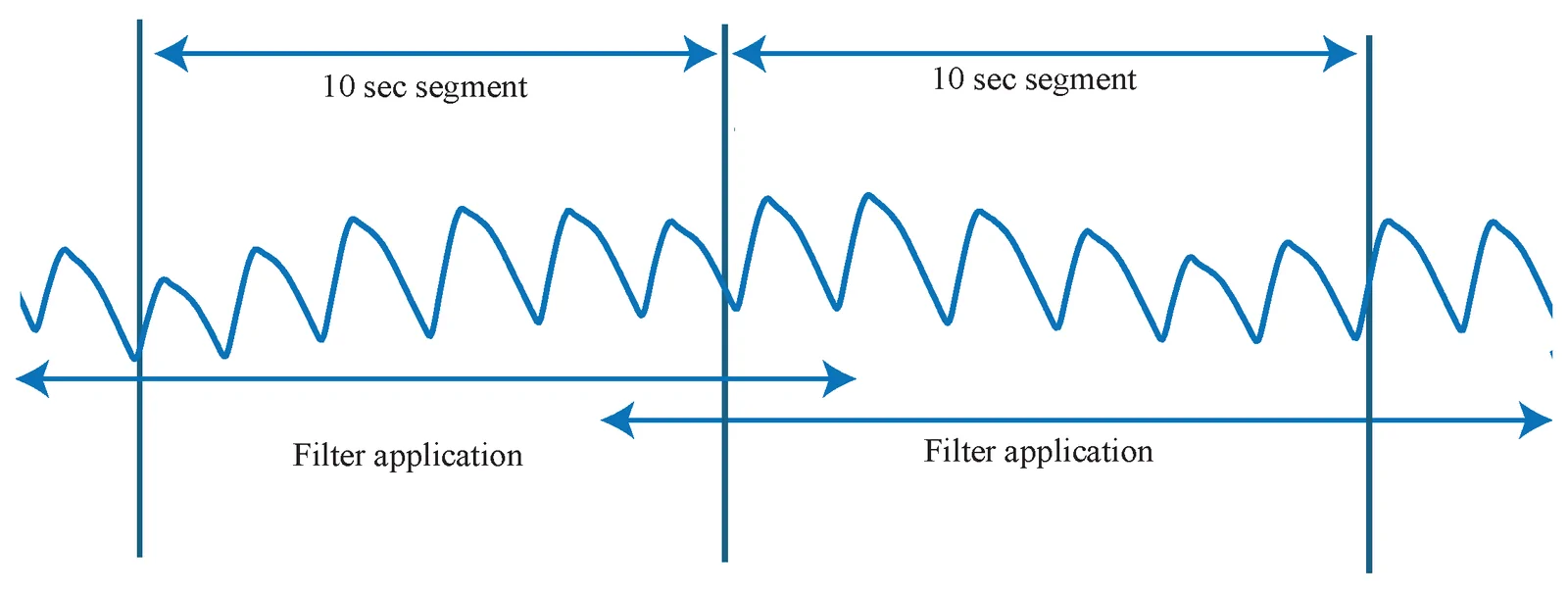
Overlap-and-blend strategy used at segment boundaries. Filtering is applied to windows that extend beyond the nominal 10 s segment, and overlapping samples are combined with linear weights to reduce edge effects and discontinuities between adjacent segments.

The filtering stage combines the motion label ${\hat{k}}_{i}$ and the reliability flag $r_{i}$. Each 10 s window is filtered according to its own label, using an extended window that includes neighboring signal on both sides, and the outputs of adjacent windows are blended linearly, as shown in Figure 10 and detailed below.

A five-level discrete wavelet transform (DWT) with the Symlet 4 (sym4) wavelet is applied to each extended window [34]. At a sampling rate of $f_{s}=55$ Hz, the approximate dyadic frequency bands of the detail coefficients are shown in Table 4. These bands are approximate because real wavelet filters are not ideal brick-wall filters, but they are useful for identifying coefficients that overlap the expected PPG pulsatile range.

The filtering rule avoids zeroing $D_{4}$ and $D_{5}$, because these bands can contain physiological pulsatility. This design choice follows the same principle as recent reconstruction and completion studies: artifact attenuation is useful only if the physiological waveform needed for downstream interpretation is not unnecessarily distorted [20,35]. The approximation coefficient $A_{5}$ is removed to suppress baseline drift below approximately 0.86 Hz. Detail coefficients $D_{1}$–$D_{3}$ are soft-thresholded, with the threshold strength selected according to motion state and reliability. Let $W_{j}$ be the coefficients at level *j*, and let $\lambda_{j}=\eta_{k,r}\;\mathrm{median}(|W_{j}\left|\right)/0.6745$ for j=1,2,3, where $\eta_{k,r}$ depends on the motion cluster and on the reliability flag and was selected on the training participants only (Section 2.6). Soft-thresholding is applied as

$$T\left(W_{j},\lambda_{j}\right)=\mathrm{sign}\left(W_{j}\right)\max(|W_{j}\left|-\lambda_{j},0\right),\;j\in \left\{1,2,3\right\}.$$

The $D_{4}$ and $D_{5}$ coefficients are preserved to protect the cardiac waveform unless future validation with a clean reference supports a more aggressive rule. The signal is reconstructed using the inverse wavelet transform (waverec).

To minimize discontinuities at segment boundaries, each window is extended by 2 s on both sides of the nominal 10 s segment before the DWT is applied. The filtered output is retained over the central 10 s plus 0.5 s on each side, so that adjacent windows overlap by 1 s. In this overlap region, the two filtered estimates are combined with complementary linear weights:

$$y\left[n\right]=\alpha \left[n\right]y_{\mathrm{left}}\left[n\right]+\left(1-\alpha [n]\right)y_{\mathrm{right}}\left[n\right],\;\alpha \left[n\right]\in \left[0,1\right].$$

This overlap-and-blend step reduces edge effects and improves waveform continuity between segments with different motion or reliability labels. Windows closer than 2 s to the start or end of a continuous recording block were not analyzed.

### 2.6. Beat-Level Validation Against ECG

To assess beat-detection and timing performance, the filtered PPG was compared with the simultaneously recorded Polar H10 ECG. Only time intervals in which both devices were recording were analyzed. ECG R peaks were detected after zero-phase band-pass filtering (second-order Butterworth, 5–20 Hz) using an adaptive amplitude threshold and a minimum inter-peak distance of 0.3 s, following the principles of classical QRS detectors [36]. A 10 s window was considered to have a valid ECG reference if it contained at least four R peaks, all RR intervals were between 0.3 and 2.0 s, and the coefficient of variation of the RR intervals was below 0.3. The ECG provides an independent temporal reference, but not an independent reference for PPG pulse morphology.

To avoid fitting and evaluating the method on the same data, the 50 recordings were randomly split, with a fixed random seed, into a training set of 10 recordings and a test set of 40 recordings. The split was performed at participant level, and no participant contributed to both sets. Recordings were excluded if they lacked PPG data or the ECG device or had unreadable timestamps, and only the portions of each recording with temporal overlap between the two devices were analyzed. In addition, four files were found to contain recordings identical to those of other files, owing to an error in the preparation of the data files; each of these recordings was included only once. The resulting data flow is summarized in Table 5. A small training set and a large test set were deliberately chosen because the method has few parameters to estimate, whereas reliable participant-level statistical comparisons require a large number of held-out participants.

The cluster centroids, the Min–Max normalization parameters, the CQI normalization bounds (1st and 99th percentiles of $L_{i}$ over the training windows), and the threshold multipliers $\eta_{k,r}$ were estimated on the training recordings only and applied unchanged to the test recordings. For each combination of motion cluster *k* and reliability flag *r*, $\eta_{k,r}$ was selected from the grid {0.5,1,1.5,2,3,5,10} as the value minimizing the IBI error defined below, with missed beats counted as full errors; the per-participant median error was computed first and then the median across training participants was minimized. The selected values are reported in Table 6, and all other model parameters are listed in Table 7.

The proposed method was compared with three reference methods and with the unfiltered signal: (i) a second-order Butterworth band-pass filter (0.5–3 Hz) applied with zero-phase filtering (filtfilt); and (ii) a fixed five-level Daubechies 4 (Db4) wavelet filter in which $A_{5}$ was removed and $D_{1}$–$D_{3}$ were soft-thresholded with the universal threshold $\lambda_{j}=\sigma_{j}\sqrt{2\ln N}$, where $\sigma_{j}=\mathrm{median}(|W_{j}\left|\right)/0.6745$ was estimated separately at each level and *N* is the window length; and (iii) an accelerometer-referenced adaptive noise canceller based on the normalized least-mean-squares (NLMS) algorithm [37], a widely used approach for motion-artifact reduction in wearable PPG [12,17]. In the NLMS filter, the PPG and the three accelerometer axes were band-pass filtered (second-order Butterworth, zero-phase) and each axis was scaled to unit variance; a multichannel finite-impulse-response filter with *M* taps per axis then estimated the motion-correlated component of the PPG, which was subtracted from it. The weights were updated with step $\mu /(\delta +∥u\left[n\right]{∥}^{2})$, where u[n] is the vector of the last *M* samples of the three axes and δ=0.01·3M. For a fair comparison, the pre-filtering band (0.5–3 or 0.5–5 Hz), M∈{8,16}, and $\mu \in \{10^{-4},5\cdot 10^{-4},10^{-3},5\cdot 10^{-3},10^{-2}\}$ were selected on the training recordings with the same objective used for $\eta_{k,r}$. The selected configuration was 0.5–5 Hz, M=16, and $\mu =10^{-4}$.

In each 10 s window, PPG peaks were detected as local maxima separated by at least 0.33 s and with a prominence of at least 0.3 times the interquartile range of the window, and their agreement with the ECG was assessed as in recent PPG beat-detection benchmarks [38]. Each R peak was matched to the first PPG peak occurring between 0.05 s and min(0.8s,0.9RR) after it. The following metrics were computed:

- Beat-detection sensitivity (Se) and positive predictive value (PPV) of the PPG peaks with respect to the R peaks.
- Inter-beat interval (IBI) error: mean absolute difference between consecutive matched PPG peak intervals and the corresponding RR intervals. It was computed both on detected beats only and with missed beats counted as full errors (error equal to the RR interval).
- HR error: absolute difference between the HR derived from the median PPG peak interval and from the median RR interval of the window.
- Peak-timing precision: standard deviation of the delay between each R peak and its matched PPG peak. The absolute delay was not used because it includes the pulse arrival time and a possible clock offset between the two devices.
- Peak amplitude ratio: peak-to-trough amplitude of each filtered beat divided by that of the corresponding linearly detrended unfiltered beat, with beats delimited by consecutive R peaks and resampled to 100 points.
- Pulse-to-pulse waveform correlation: median correlation between each beat and the mean beat of the window.
- Segment-level signal quality: skewness [39] of each window before and after filtering.

For each participant and method, the median of each metric across windows was computed, and methods were compared with the two-sided Wilcoxon signed-rank test at the participant level. The 95% confidence interval (CI) of the median paired difference between each reference method and the proposed method was obtained by bootstrap resampling of participants (2000 resamples). The analysis was also stratified by motion cluster. Because multiple methods, metrics, and motion strata were examined without multiplicity correction, the reported *p* values are interpreted as exploratory.

## 3. Results

### 3.1. Clustering Results

**Figure 11:**
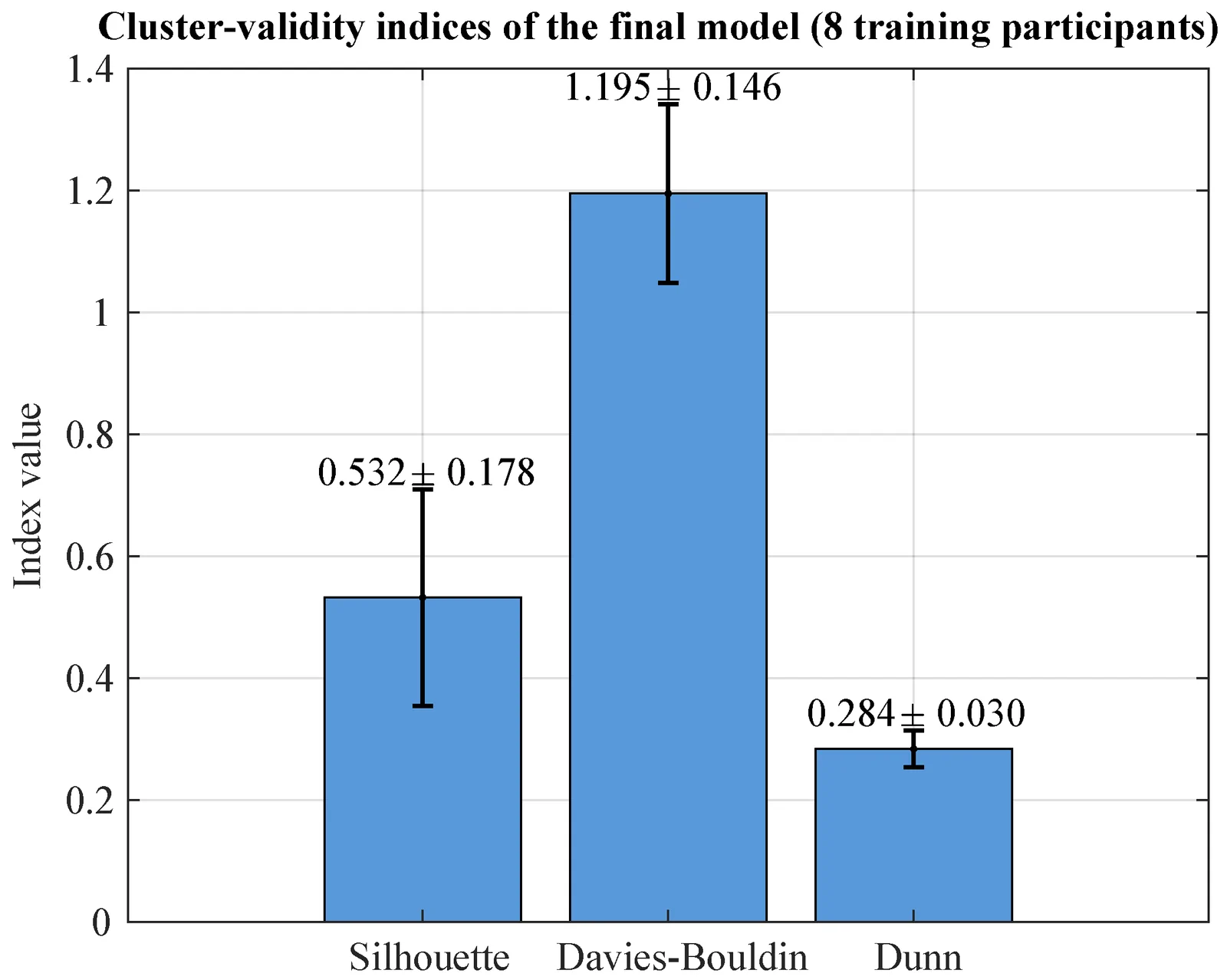
Clustering performance metrics for the selected standard-deviation–skewness feature pair. Bars show the mean value across the eight training participants, and error bars and annotations report mean ± standard deviation for the Silhouette, Davies–Bouldin, and Dunn indices.

The cluster-validity indices of the final model were computed separately for each of the eight training participants. With k=3, the Silhouette Mean was 0.532±0.178, the Davies–Bouldin Index was 1.195±0.146, and the Dunn Index was 0.284±0.030 (mean ± standard deviation across participants). These values indicate moderately separated clusters rather than sharply distinct groups, which is expected because daily-life movement intensity varies along a continuum. Figure 11 summarizes these clustering performance metrics.

**Figure 12:**
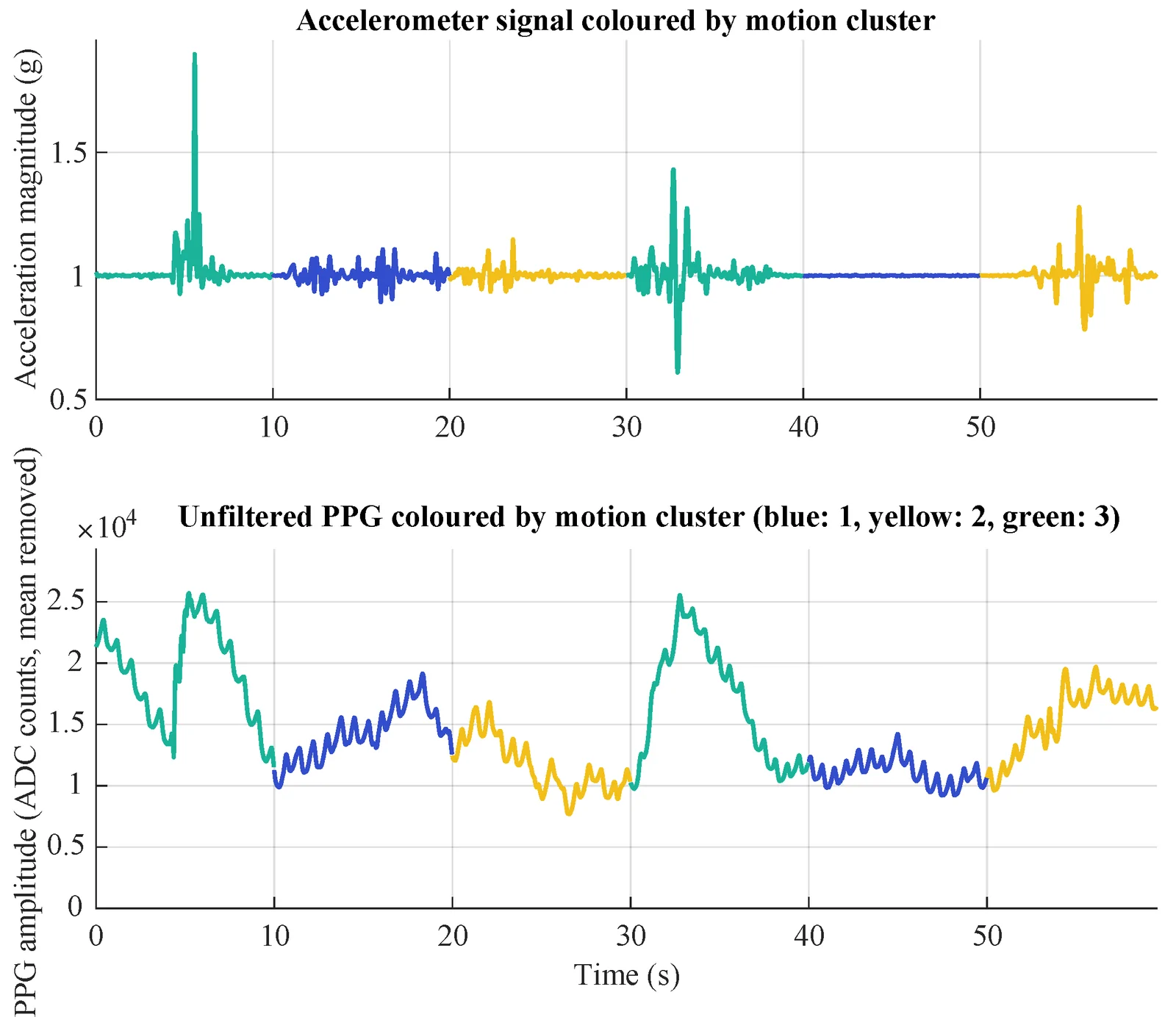
Motion-state labels mapped from acceleration to synchronized PPG segments. The upper trace shows the acceleration magnitude (g) colored by cluster, and the lower trace shows the corresponding unfiltered PPG (mean removed) with the same motion-state labels: blue, cluster 1 (rest/low motion); yellow, cluster 2 (moderate motion); green, cluster 3 (dynamic motion). These colors represent motion context, not direct PPG quality.

Figure 12 illustrates the motion-state segmentation of the acceleration and PPG signals. The top plot shows the acceleration magnitude over 1 min of a test recording, with colors representing rest/low-motion, moderate-motion, and dynamic-motion states. The bottom plot maps the same labels onto the corresponding unfiltered PPG segments. The labels should be interpreted as motion states rather than as direct PPG quality categories; contact quality is represented separately by the CQI.

#### Quality-Flagging Results

**Figure 13:**
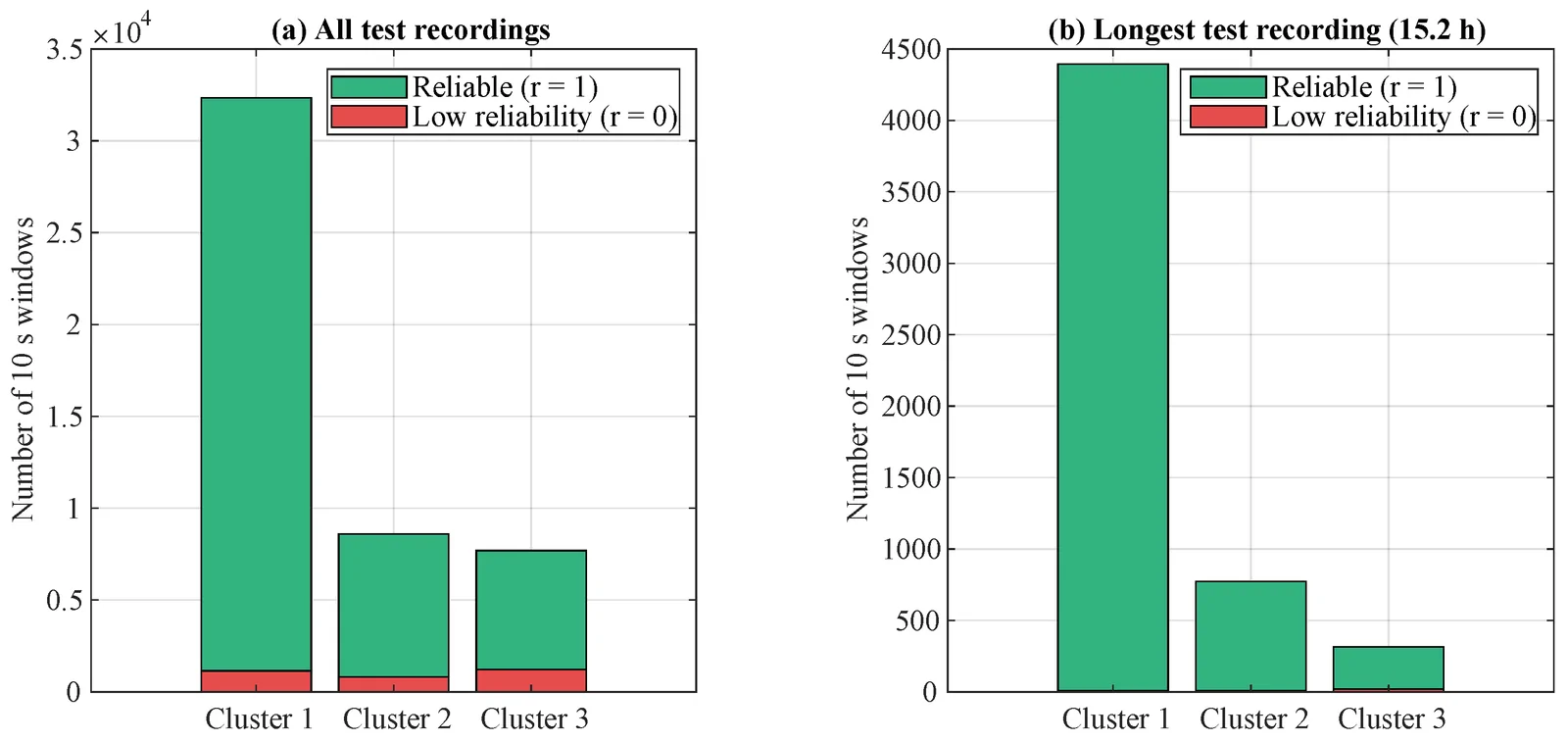
Reliability breakdown within each motion-state cluster. Panel (**a**) shows all test recordings, and panel (**b**) shows the longest test recording. Colors indicate the CQI-based reliability flag within each fixed motion cluster. Low-CQI segments are flagged as low reliability rather than reinterpreted as a separate motion state.

The CQI identified segments in which ambient-light behavior suggested low contact reliability. Figure 13 shows the reliability breakdown within each fixed motion cluster for all test recordings and for the longest test recording. The displayed counts stratify the original motion-cluster assignments by CQI-based reliability; low-CQI windows are flagged as low reliability rather than being reassigned to a different motion state.

The proportion of low-reliability segments reflects intervals in which ambient-light artifacts or poor sensor contact were detected. Because the CQI is separate from motion clustering, this proportion is not interpreted as evidence of increased movement; rather, it indicates the fraction of windows requiring quality-aware handling.

### 3.2. Filtering Results

**Figure 14:**
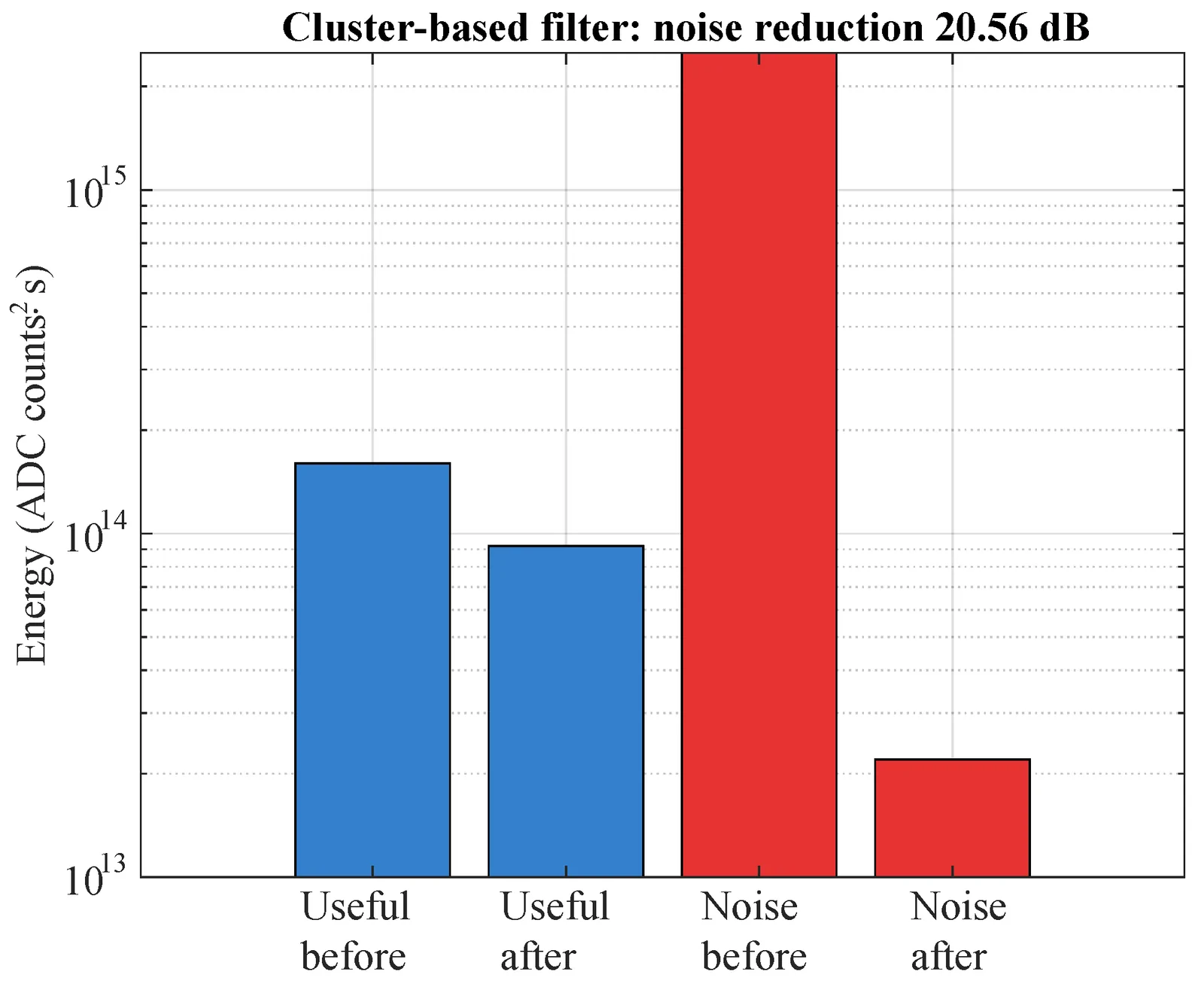
Useful-band (0.5–3 Hz) and out-of-band (noise) spectral energies before and after cluster-based filtering, pooled over the 27 test participants (logarithmic scale). The pooled noise-energy reduction is 20.6 dB; participant-level medians and interquartile ranges are reported in Table 8.

Filtering performance was quantified by integrating the PSD over predefined useful and noise frequency regions. For the analysis in Figure 14, the useful band was defined as 0.5–3.0 Hz, corresponding to the main heart-rate range considered in this study, and the noise region was defined as the complementary evaluated spectrum outside that band. The relative spectral energy in a band was computed as

$$E_{\left[f_{1},f_{2}\right]}=\int_{f_{1}}^{f_{2}}P_{xx}\left(f\right)\;df,$$

and was used as a relative quantity for within-recording comparisons. The noise reduction in decibels was computed as

$$\Delta E_{\mathrm{noise}}=10\log_{10}\left(\frac{E_{\mathrm{noise},\mathrm{before}}}{E_{\mathrm{noise},\mathrm{after}}}\right).$$

The band energies were computed for each test participant over all analyzed windows, and the results are summarized as median and interquartile range (IQR) across the 27 test participants (Table 8). For the proposed method, the median out-of-band noise-energy reduction was 27.7 dB (IQR 22.0–35.0 dB). When the energies of all participants were pooled (Figure 14), the reduction was 20.6 dB; the pooled value is lower because it is dominated by the participants with the largest noise energy.

The useful-band spectral energy decreased by a median of 41.0% (IQR 33.1–52.2%). A large part of this reduction is expected from the design of the filter, because the removed approximation band $A_{5}$ (0–0.86 Hz) overlaps the lower part of the 0.5–3.0 Hz useful band, which contains both baseline drift and, at low heart rates, pulsatile energy. Therefore, noise suppression should be considered together with useful-band preservation. Based on the same partition, the median SNR changed from −16.0 dB (IQR −19.6 to −10.0 dB) before filtering to 9.5 dB (IQR 6.8–13.1 dB) after filtering, corresponding to a median SNR improvement of 25.2 dB (IQR 19.9–32.4 dB). Table 8 reports the same measures for the reference methods.

**Figure 15:**
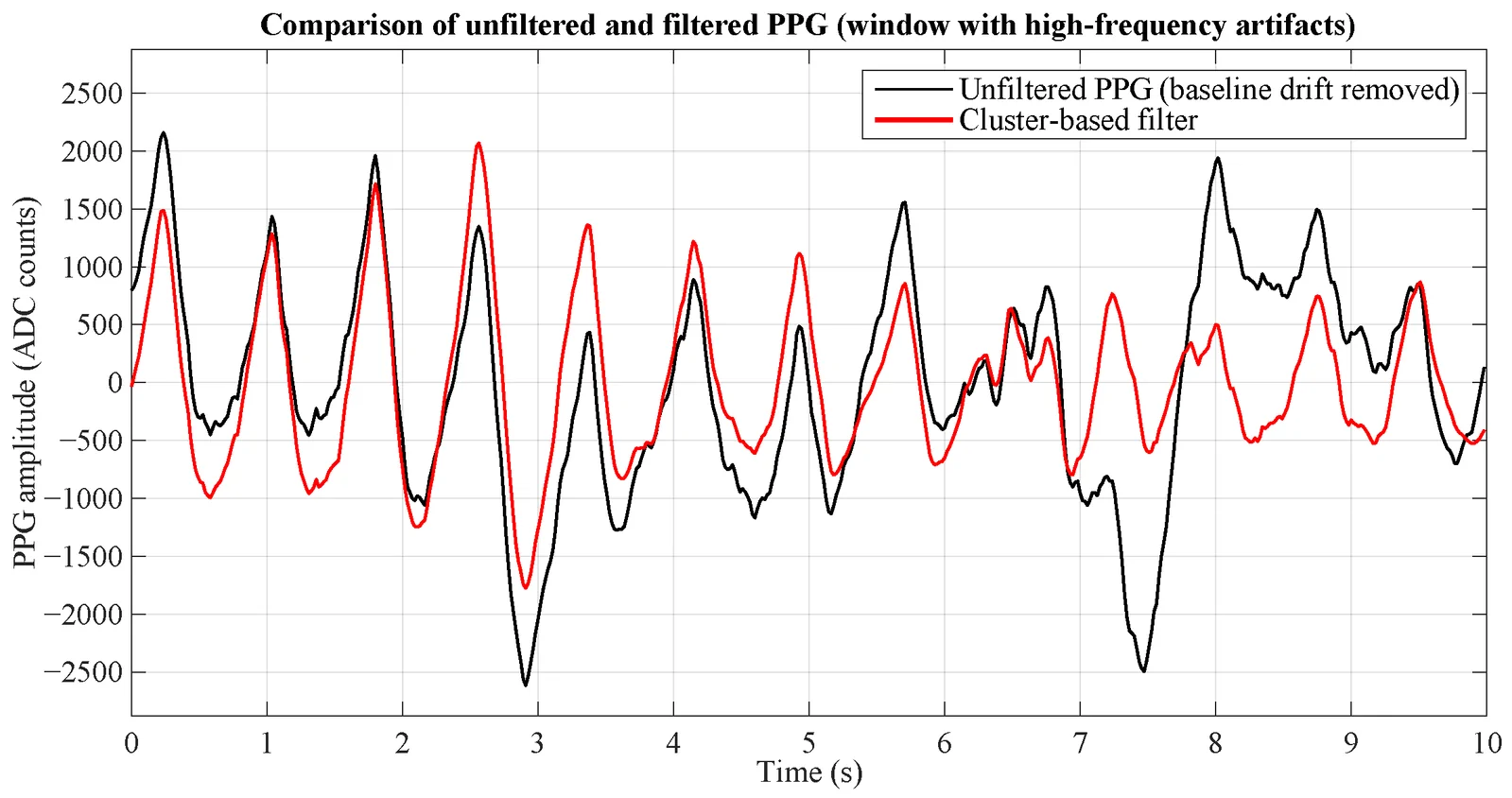
Example of cluster-based filtering in a window with high-frequency artifacts, selected at the 90th percentile of the high-frequency (>5 Hz) power fraction of the unfiltered PPG among reliable test windows with a valid ECG. The unfiltered PPG (black) is shown after removing the slow baseline drift (2 s moving average) for display only; the filtered PPG is shown in red. Amplitudes are in raw ADC counts.

These values describe the selected band-energy definition and should not be interpreted as proof of beat-level performance; beat-detection and timing are assessed against the ECG reference in Section 3.3. A time-domain example in a window with high-frequency artifacts is shown in Figure 15.

**Figure 16:**
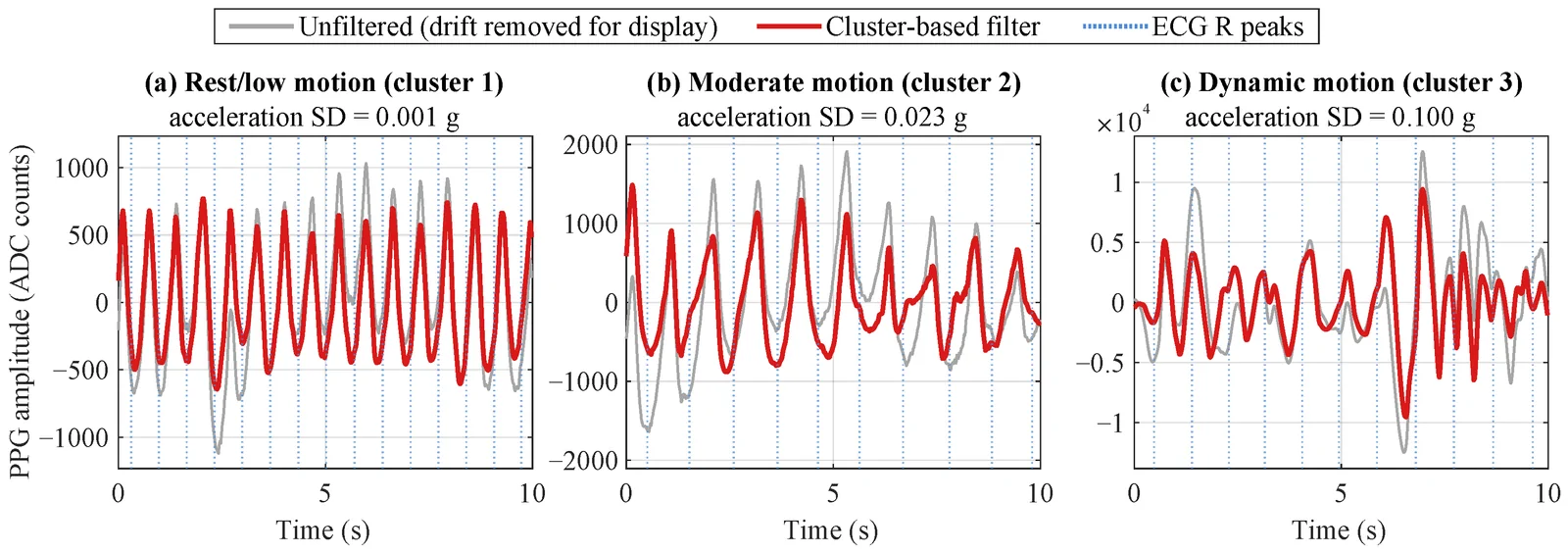
Filtering examples for the three accelerometer-derived motion states: (**a**) rest/low motion, (**b**) moderate motion, and (**c**) dynamic motion. Each window is the reliable test window with the median acceleration standard deviation of its cluster, which is reported above each panel. The unfiltered PPG (grey) is shown after removing the slow baseline drift for display only, the filtered PPG in red, and ECG R peaks as dotted lines. Amplitudes are in raw ADC counts.

Figure 16 shows examples from the three motion states, each selected as the reliable test window with the median acceleration standard deviation of its cluster. In these segments, the filter attenuates high-frequency disturbances while the filtered pulses remain aligned with the ECG R peaks. Quantitative beat-level results against the ECG reference are reported in Section 3.3.

### 3.3. Beat-Level Validation Results

The test set comprised 27 participants and 48,641 ten-second windows (approximately 135.1 h) of synchronous ECG and PPG, of which 48,079 (98.8%) had a valid ECG reference. Of all test windows, 66.5% were assigned to cluster 1, 17.7% to cluster 2, and 15.8% to cluster 3, and 6.6% were flagged as low reliability (r=0). The proportion of low-reliability windows increased with motion: 1149 (3.6%), 824 (9.6%), and 1217 (15.8%) windows in clusters 1, 2, and 3, respectively. Table 9 summarizes the participant-level results.

Compared with band-pass filtering, the proposed method detected more beats (median Se 1.00 vs. 0.91, p<0.001) and reduced the IBI error when missed beats were included (128.0 vs. 181.2 ms, p<0.001), but with a lower PPV (0.92 vs. 1.00, p=0.005), indicating that a fraction of the additional PPG peaks did not correspond to an R peak. On detected beats, however, band-pass filtering provided more precise timing (IBI error 24.6 vs. 38.7 ms, p=0.005; peak-timing SD 39.3 vs. 49.9 ms, p=0.009) and a higher pulse-to-pulse correlation (0.975 vs. 0.948, p<0.001), whereas the HR error did not differ significantly (0.90 vs. 1.27 bpm, p=0.98). Compared with the fixed Db4 filter, the proposed method yielded lower IBI error on detected beats (38.7 vs. 41.3 ms) and with missed beats included (128.0 vs. 160.8 ms), lower HR error (1.27 vs. 1.56 bpm), and lower peak-timing SD (49.9 vs. 53.0 ms; all p≤0.004). The accelerometer-referenced NLMS filter showed a similar pattern to band-pass filtering: compared with the proposed method, it provided more precise timing on detected beats (IBI error 26.6 vs. 38.7 ms, p<0.001) and a similar HR error (0.82 vs. 1.27 bpm, p=0.09), but it detected fewer beats (median Se 0.92 vs. 1.00, p<0.001) with a higher PPV (1.00 vs. 0.92, p=0.001) and had a higher IBI error with missed beats included (156.8 vs. 128.0 ms, p=0.004; median paired difference 31.2 ms, 95% CI −0.1 to 45.9 ms). During training, the objective increased monotonically with the NLMS step size for all bands and filter orders, so the smallest step size was selected. The paired differences and their confidence intervals are reported in Table 10. The median peak amplitude ratio was 0.95 for the proposed method, 0.93 for the band-pass and Db4 filters, and 0.97 for the NLMS filter, and the median window skewness increased from 0.08 in the unfiltered signal to 0.30 after proposed filtering and 0.25 after band-pass filtering.

Table 11 reports the comparison with band-pass filtering stratified by motion cluster. In low-motion windows (cluster 1), band-pass filtering yielded a lower IBI error on detected beats (19.3 vs. 32.4 ms, p<0.001) and the HR error did not differ significantly, whereas the IBI error with missed beats included was lower with the proposed method in 16 of 27 participants (median paired difference −16.5 ms, p=0.028). In moderate- and high-motion windows (clusters 2 and 3), the proposed method detected more beats (higher Se in 24 of 27 participants in both clusters) and reduced the IBI error with missed beats included (lower in 26 and 25 of 27 participants, respectively) and the HR error (p=0.034 and p=0.019), whereas the IBI error on detected beats did not differ significantly.

## 4. Discussion

The preprocessing of wearable PPG signals is challenging because the dominant artifacts are not stationary. Motion, changes in contact pressure, and ambient light leakage can appear and disappear within short intervals, and their spectral content can overlap physiologically relevant bands. Fixed band-pass filters, such as a 0.5–3 Hz Butterworth filter, are useful references but cannot adapt their behavior to local signal quality. Their effect also depends on the implementation: a causal filter can introduce phase delay, whereas a zero-phase implementation such as filtfilt avoids phase delay at the cost of being non-causal and therefore unsuitable for strict real-time use. For this reason, the reference filter implementation must be specified when comparisons are made.

Fixed wavelet filters provide time-frequency localization, but a single threshold and a fixed set of suppressed levels can be inappropriate for long free-living recordings. The proposed method addresses this limitation by using accelerometer-derived motion states and ambient-light-derived reliability flags to adapt threshold strength. Importantly, the wavelet bands that overlap plausible cardiac components at 55 Hz, especially $D_{4}$ and $D_{5}$, are preserved rather than blindly zeroed. This design reduces the risk of removing the pulsatile signal while still suppressing baseline drift through $A_{5}$ and high-frequency artifacts through thresholding of $D_{1}$–$D_{3}$.

A key feature of the pipeline is the separation of motion state and contact quality. Accelerometer clusters describe movement-related context, whereas the CQI describes reliability of optical coupling. Treating low contact quality as a separate flag avoids assigning ambient-light artifacts to a motion cluster and makes the resulting labels easier to interpret. This distinction is especially important when the method is transferred to different participants, placements, or devices.

The recent literature reinforces this distinction. Contact-pressure studies have shown that PPG amplitude, derived morphology, SpO_2_, pulse-arrival-time features, and heart-rate accuracy can all vary with sensor–skin pressure, and that the optimal pressure range depends on the measurement objective, wavelength, and activity intensity [30,31,40]. A large wrist-PPG analysis also found that posture and sensor height relative to the heart are important determinants of PPG signal quality [32]. These findings suggest that poor optical coupling is not simply another form of movement; it is a separate measurement condition that can alter both the DC component and the pulsatile morphology. The proposed CQI should therefore be interpreted as a quality-control mechanism for the optical interface rather than as a surrogate activity label.

The motion-clustering stage is also consistent with recent artifact-aware approaches. Thakur et al. combined pre-quality checking with Hankel decomposition for HR estimation from a PPG patch and accelerometer data [19], while Hao et al. classified motion-artifact intensity before selecting a heart-rate extraction strategy [21]. The present work uses a simpler unsupervised accelerometer clustering strategy because the goal is not to reconstruct HR directly, but to choose a conservative or less aggressive denoising rule according to the recording context. This keeps the preprocessing interpretable and avoids embedding the evaluation target, such as HR, into the filtering stage.

Machine-learning and deep-learning approaches provide a complementary perspective. Recent ML-based work has reconstructed HR from PPG under motion artifacts [20], and generative models have been used to complete missing or corrupted PPG waveforms while preserving local and global waveform consistency [35]. Lightweight models such as Tiny-PPG and energy-efficient semi-supervised signal-quality assessment methods demonstrate that real-time artifact or quality detection on wearable hardware is increasingly feasible [14,16]. However, these methods also require representative training data and careful external validation. The present rule-based wavelet design is less flexible than learned reconstruction, but it is transparent, requires fewer training assumptions, and can be audited at the level of retained and attenuated frequency bands.

**Figure 17:**
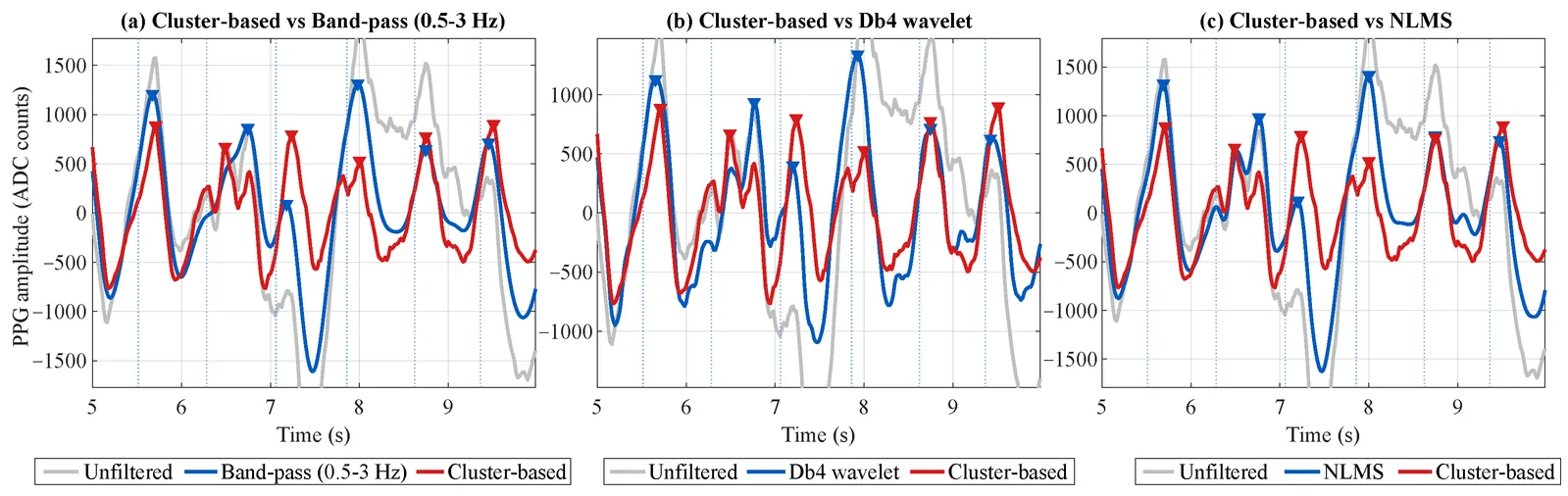
Visual comparison of filtering methods on a 5 s excerpt of the window of Figure 15, corresponding to the half of the window in which the methods differed most. The cluster-based filter (red) is compared with (**a**) the Butterworth band-pass filter (0.5–3 Hz), (**b**) the fixed Daubechies 4 wavelet filter, and (**c**) the NLMS adaptive filter (blue). The unfiltered PPG (light grey) is shown after removing the slow baseline drift for display only. Triangles mark the peaks detected with the same detector used in the beat-level validation, and dotted lines mark the ECG R peaks. Amplitudes are in raw ADC counts.

Figure 17 provides a visual comparison between the proposed approach and the three reference methods. The figure is intended as an illustrative comparison of waveform behavior rather than as a complete statistical benchmark.

**Figure 18:**
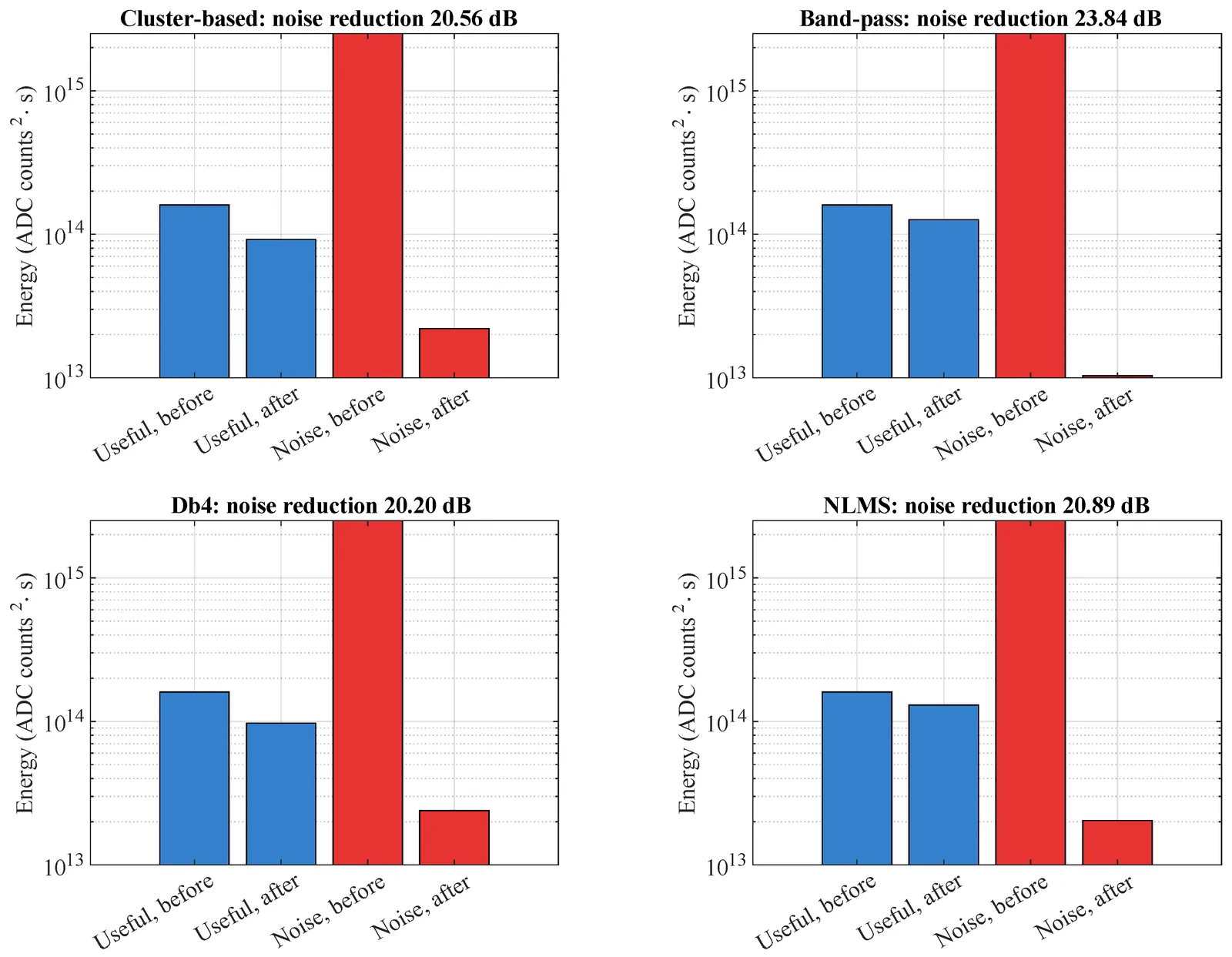
Band-energy results of the proposed method and the reference methods: useful-band and out-of-band (noise) spectral energies before and after filtering, pooled over the 27 test participants (logarithmic scale). The pooled noise-energy reductions are 20.6 dB for the proposed method, 23.8 dB for the band-pass filter, 20.2 dB for the Db4 filter, and 20.9 dB for the NLMS filter; participant-level medians and interquartile ranges are reported in Table 8.

Figure 18 and Table 8 compare band-energy changes for the proposed filter and the three reference methods. The median out-of-band noise-energy reductions were 27.7 dB for the proposed method, 29.4 dB for the band-pass filter, 27.4 dB for the Db4 filter, and 27.2 dB for the NLMS filter, with median useful-band energy changes of −41.0%, −20.2%, −38.4%, and −17.9%, respectively (Table 8). The band-pass reference achieved the largest reduction because its passband coincides with the useful band used in the evaluation, and both the band-pass and NLMS filters preserved more useful-band energy than the wavelet-based methods, whose removal of $A_{5}$ attenuates the lowest part of the useful band. Because these band-energy measures cannot show whether individual beats are preserved, the methods were also compared at the beat level against the ECG reference.

The beat-level validation shows a trade-off rather than a uniform advantage of any single method. In low-motion windows, which represented about two thirds of the test data, band-pass filtering provided the most precise peak timing on detected beats. The 0.5–3 Hz band-pass filter produces a smooth, nearly sinusoidal waveform with sharp peaks, which favors peak timing but removes waveform components above 3 Hz. In moderate- and high-motion windows, the proposed method, which preserves $D_{4}$ and $D_{5}$ and attenuates rather than removes $D_{1}$–$D_{3}$, recovered substantially more beats, which reduced the IBI error when missed beats were considered and reduced the HR error during movement. This gain in sensitivity was accompanied by a lower PPV, which was also observed for the Db4 filter and may reflect secondary waveform peaks, such as those associated with the dicrotic notch, that are retained when the components above 3 Hz are preserved. In addition, because the threshold multipliers were selected to minimize the IBI error with missed beats counted as full errors, the training objective favored sensitivity over positive predictive value, whereas the band-pass and Db4 references were not optimized. The accelerometer-referenced NLMS filter behaved similarly to band-pass filtering. Because the selected step size was the smallest in the grid, the adaptive filter operated close to a fixed 0.5–5 Hz band-pass filter, indicating that accelerometer-referenced cancellation provided little benefit in these recordings. Adaptive noise cancellation is most effective when motion artifacts are strong and correlated with the accelerometer, such as during running or periodic arm movement [12,17], whereas the present recordings mainly contain low-intensity daily activities. The proposed method also outperformed the fixed Db4 wavelet filter on all timing metrics. However, the selected multipliers $\eta_{k,r}$ were small and similar across clusters (0.5–1.0), whereas the universal threshold of the Db4 reference corresponds to approximately 3.6 times the noise estimate at each level. The advantage over the Db4 reference therefore appears to derive mainly from the lighter, training-selected thresholding and from the choice of retained bands, rather than from cluster-specific adaptation of the threshold, which did not provide a measurable additional benefit in this dataset. These results also suggest that the motion context could be used to select the type of filter, for example band-pass filtering during rest and wavelet-based filtering during movement; because this hypothesis was generated from the present results, it should be tested prospectively on independent data.

The 0.5–3.0 Hz useful band used in the band-energy analysis and in the band-pass reference filter corresponds to heart rates of 30–180 bpm, which covers the range expected in this cohort during rest and daily activities (mean heart rate 77 bpm). The proposed filter is not restricted to this band, because it retains the $D_{4}$ and $D_{5}$ bands (approximately 0.86–3.44 Hz) and attenuates rather than removes higher-frequency components. However, the removal of the approximation band $A_{5}$ (0–0.86 Hz), which suppresses baseline drift, also attenuates the fundamental component of heart rates below approximately 52 bpm. Such heart rates may occur during sleep or in patients treated with beta-blockers, who represented 17% of the participants with clinical data (Table 1). The current configuration is therefore best suited to heart rates above approximately 52 bpm, and adapting the lowest retained band to the expected heart-rate range of each patient would be a natural extension.

The clustering metrics indicate a moderate separation of accelerometer windows in the selected feature space, with a Silhouette Mean of 0.532±0.178, a Davies–Bouldin Index of 1.195±0.146, and a Dunn Index of 0.284±0.030, rather than a sharp separation. Because its absolute magnitude depends on feature scaling and cluster diameters, the Dunn Index should be interpreted relative to alternative feature sets rather than as standalone proof of well-defined boundaries. Similarly, the median noise-energy reduction of 27.7 dB and SNR improvement of 25.2 dB are meaningful under the defined band-energy partition, but they do not by themselves establish preservation of all clinically relevant waveform features.

Several limitations remain. First, the numerical centroids, normalization parameters, and thresholds reported in Table 7 are dataset-specific and should be re-estimated or validated when the method is applied to other devices or cohorts. Second, the CQI is a heuristic indicator based only on the ambient-light channel: it has not been validated against independent contact-quality labels, it cannot distinguish reduced contact from light penetration with stable contact, and its threshold of 0.5 was used as an initial operating point. Third, although beat-level agreement was assessed against ECG, the ECG and PPG were acquired by two devices whose clocks were aligned through the acquisition application, so absolute pulse arrival times were not analyzed; the ECG does not provide an independent reference for PPG pulse morphology; the training set included only eight participants, which increases the risk of dataset-specific parameter selection; the proposed method showed lower peak-timing precision than band-pass filtering on detected beats; and the averaging of the three PPG channels was compared only qualitatively with SNR-weighted fusion, whereas best-channel selection and a quantitative comparison of fusion strategies were not evaluated; in addition, removing $A_{5}$ attenuates heart rates below approximately 52 bpm. The statistical comparisons were not adjusted for multiplicity and should therefore be interpreted as exploratory. Finally, the recordings did not include structured exercise, so severely corrupted segments such as those produced by running were not represented; the performance of the method under such conditions remains untested, and the analysis should be extended to other cohorts, higher-intensity activities, additional device placements, and real-time implementations before general claims about robustness can be made.

Future comparisons should include recent quality-assessment and artifact-detection baselines, including SQI-guided filtering and reference-signal-less ML methods summarized in recent reviews [15]; these approaches require labeled training data that were not available in this study. Such comparisons should report not only HR error or aggregate SNR, but also signal-quality classification performance, morphology-sensitive metrics, and computational cost. This broader evaluation would clarify whether the proposed CQI and cluster-based wavelet rules are best used as a standalone preprocessing method, as a front-end quality gate before ML reconstruction, or as a lightweight fallback when embedded resources or labeled training data are limited.

## 5. Conclusions

This study presents an adaptive preprocessing pipeline for wearable PPG signals that integrates accelerometer-based motion-state clustering, ambient-light-based contact-quality assessment, and cluster-based wavelet filtering. The formulation defines the CQI on a consistent 0–1 scale, handles degenerate ambient-light segments explicitly, separates motion labels from contact-quality flags, and preserves wavelet detail bands that overlap the expected cardiac frequency range.

In the 27 held-out participants, the pipeline reduced out-of-band noise energy by a median of 27.7 dB and improved the band-energy SNR by a median of 25.2 dB, while the useful-band energy decreased by a median of 41.0%, more than with band-pass or NLMS filtering. These results indicate that the method can substantially attenuate artifacts, but they also show why noise reduction and useful-signal preservation must be reported together. In a beat-level validation against ECG in 27 participants not used for model fitting, the proposed method detected more beats than band-pass filtering and reduced the IBI error when missed beats were considered, particularly during movement, whereas band-pass filtering provided more precise peak timing at rest and on detected beats. An accelerometer-referenced NLMS filter showed the same trade-off as band-pass filtering, and its training selected minimal adaptation, suggesting limited benefit of adaptive cancellation for low-intensity daily-life motion. The proposed method outperformed a fixed Db4 wavelet filter on all timing metrics, although this advantage appears to derive mainly from lighter thresholding rather than from cluster-specific adaptation.

Future work should validate the trained parameters on other devices and cohorts, validate the CQI against external contact-quality annotations, extend the beat-level validation to other cohorts and higher-intensity activities, and evaluate whether the motion context should select the type of filter rather than only the threshold strength. Recent wearable studies indicate that contact pressure, posture, activity level, and device placement should be included explicitly in these validation protocols [31,32]. Real-time deployment on wearable devices will also require computational optimization and validation under causal filtering constraints, ideally against lightweight signal-quality and artifact-detection baselines [14,15,16]. 

## Figures and Tables

**Table 1:** Characteristics of the participants with available clinical data and of the analyzed participants (training and test sets) with available clinical data; clinical data were missing for 2 of the 35 analyzed participants. Continuous variables are reported as mean ± standard deviation and categorical variables as *n* (%); when data were missing for some participants, the denominator is given. Blood pressure and heart rate were measured at enrollment. ^a^ Blood pressure ≥140/90 mmHg or treatment with antihypertensive medication (diuretics, ACE inhibitors, angiotensin receptor blockers, calcium channel blockers, or beta-blockers).

Characteristic	With Clinical Data (n=46)	Analyzed (n=33)
Age (years)	54.2 ± 15.0	53.9 ± 15.5
Women, *n* (%)	18 (39)	11 (33)
Body mass index (kg/m^2^)	27.9 ± 6.2	27.4 ± 5.4
Overweight or obese (≥25 kg/m^2^), *n* (%)	27 (59)	19 (58)
Obese (≥30 kg/m^2^), *n* (%)	15 (33)	10 (30)
Systolic blood pressure (mmHg)	131.1 ± 20.1	133.5 ± 17.8
Diastolic blood pressure (mmHg)	78.6 ± 11.3	80.1 ± 10.0
Heart rate (bpm)	77.3 ± 14.5	76.7 ± 14.4
Hypertension ^a^, *n* (%)	31 (67)	20 (61)
Diabetes, *n* (%)	8/37 (22)	2/26 (8)
Heart failure, *n* (%)	8 (17)	4 (12)
Previous stroke, *n* (%)	7 (15)	4 (12)
Sleep apnea, *n* (%)	10/45 (22)	6/32 (19)
Beta-blockers, *n* (%)	8 (17)	4 (12)
Statins, *n* (%)	16/41 (39)	12/30 (40)
Current smokers, *n* (%)	7 (15)	4 (12)
Regular alcohol consumption, *n* (%)	4 (9)	3 (9)
Regular physical activity, *n* (%)	25 (54)	19 (58)

**Table 2:** Statistical features computed from each 10 s acceleration-magnitude window and evaluated for clustering.

No.	Feature	Formula
1	Mean (μ)	$\mu =\frac{1}{N}\sum_{i=1}^{N}x_{i}$
2	Standard Deviation (σ)	$\sigma =\sqrt{\frac{1}{N-1}\sum_{i=1}^{N}{\left(x_{i}-\mu \right)}^{2}}$
3	Maximum Intensity ($x_{\max}$)	$x_{\max}=\max\left(x_{1},x_{2},\ldots ,x_{N}\right)$
4	Energy (*E*)	$E=\sum_{i=1}^{N}x_{i}^{2}$
5	Skewness ($\gamma_{1}$)	$\gamma_{1}=\frac{\frac{1}{N}\sum_{i=1}^{N}{\left(x_{i}-\mu \right)}^{3}}{\sigma^{3}}$
6	Excess Kurtosis ($\gamma_{2}$)	$\gamma_{2}=\frac{\frac{1}{N}\sum_{i=1}^{N}{\left(x_{i}-\mu \right)}^{4}}{\sigma^{4}}-3$
7	Entropy (*H*)	$H=-\sum_{b=1}^{B}p_{b}\log_{2}p_{b}$

**Table 3:** Cluster evaluation indices used during feature-pair selection.

Index	Definition
Silhouette Mean	$S=\frac{1}{N}\sum_{i=1}^{N}\frac{b(i)-a(i)}{\max\{a(i),b(i)\}}$, where a(i) is the mean distance from sample *i* to samples in its own cluster and b(i) is the lowest mean distance from sample *i* to samples in any other cluster. Higher values are better.
Davies–Bouldin Index	$DB=\frac{1}{K}\sum_{k=1}^{K}\max_{l\neq k}\left(\frac{s_{k}+s_{l}}{d(c_{k},c_{l})}\right)$, where $s_{k}$ is the mean within-cluster distance for cluster *k*, $c_{k}$ is its centroid, and *K* is the number of clusters. Lower values are better.
Dunn Index	$D=\frac{\min_{k<l}d\left(c_{k},c_{l}\right)}{\max_{m}\delta \left(m\right)}$, where δ(m) is the diameter of cluster *m*. Higher values are better and should be interpreted relative to alternative feature sets.

**Table 4:** Approximate DWT frequency bands for a 55 Hz PPG signal. Detail bands $D_{4}$ and $D_{5}$ overlap plausible heart-rate components and are therefore not blindly zeroed.

Coefficient	Approx. Band at $f_{s}=55$ Hz	Filtering Role
$D_{1}$	13.75–27.50 Hz	High-frequency sensor noise
$D_{2}$	6.88–13.75 Hz	High-frequency motion/sensor noise
$D_{3}$	3.44–6.88 Hz	Upper-frequency artifact band
$D_{4}$	1.72–3.44 Hz	Upper cardiac and possible artifact overlap
$D_{5}$	0.86–1.72 Hz	Cardiac-frequency band
$A_{5}$	0–0.86 Hz	Baseline drift and very low-frequency trends

**Table 5:** Data flow for the training and test sets. The split was performed at the participant level, so no participant contributed to both sets.

	Training Set	Test Set
Recordings assigned	10	40
Excluded: no PPG data	2	5
Excluded: no ECG device	0	2
Excluded: unreadable timestamps	0	2
Excluded: duplicate of another file	0	4
Recordings analyzed (participants)	8	27
10 s windows with synchronous ECG and PPG	13,167	48,641
Duration (h)	36.6	135.1

**Table 6:** Threshold multipliers $\eta_{k,r}$ selected on the training recordings. The number of training windows used for each selection is given in parentheses.

	Cluster 1	Cluster 2	Cluster 3
r=1 (reliable)	1.0 (6722)	0.5 (2127)	0.5 (2087)
r=0 (low reliability)	0.5 (327)	1.0 (376)	1.0 (919)

**Table 7:** Parameters estimated on the training recordings, or fixed a priori, and applied unchanged to the test recordings.

Parameter	Value
Min–Max bounds, standard deviation of acceleration magnitude	$z_{\min}=0.000$ g, $z_{\max}=0.159$ g
Min–Max bounds, skewness of acceleration magnitude	$z_{\min}=-1.222$, $z_{\max}=1.730$
Centroid $c_{1}$ (rest/low motion), normalized (SD, skewness)	(0.050, 0.378)
Centroid $c_{2}$ (moderate motion), normalized (SD, skewness)	(0.169, 0.699)
Centroid $c_{3}$ (dynamic motion), normalized (SD, skewness)	(0.604, 0.594)
CQI normalization bounds $L_{\min}$, $L_{\max}$	6.504, 22.390
CQI degenerate-case parameters $\epsilon_{A}$, $\epsilon_{v}$, $r_{\mathrm{sat}}$	0, 1 (squared raw digital units), 0.5
CQI threshold $T_{q}$	0.5
Threshold multipliers $\eta_{k,r}$	Table 6
Window extension and overlap for blending	2 s on each side; 1 s overlap
NLMS reference filter	0.5–5 Hz, M=16, $\mu =10^{-4}$

**Table 8:** Band-energy results in the 27 test participants: median [interquartile range] across participants. The SNR before filtering was −16.0 dB [−19.6, −10.0] for all methods.

Method	Noise-Energy Reduction (dB)	Useful-Band Energy Change (%)	SNR After Filtering (dB)	SNR Improvement (dB)
Proposed	27.7 [22.0, 35.0]	−41.0 [−52.2, −33.1]	9.5 [6.8, 13.1]	25.2 [19.9, 32.4]
Band-pass	29.4 [24.8, 33.5]	−20.2 [−21.6, −17.0]	12.1 [10.9, 13.1]	28.2 [24.0, 32.9]
Db4	27.4 [21.9, 35.0]	−38.4 [−48.7, −35.1]	9.3 [6.4, 13.1]	25.3 [20.1, 32.2]
NLMS	27.2 [21.9, 31.5]	−17.9 [−21.6, −15.4]	9.6 [9.0, 10.4]	26.1 [21.2, 30.6]

**Table 9:** Beat-level validation against ECG in the 27 test participants. Values are medians across participants of the per-participant median of each metric. * Computed only on the small fraction of beats detected in the unfiltered signal and therefore not comparable with the filtered signals.

Metric	Proposed	Band-Pass	Db4	NLMS	Unfiltered
Se	1.00	0.91	1.00	0.92	0.33
PPV	0.92	1.00	0.92	1.00	1.00 *
IBI error, missed beats included (ms)	128.0	181.2	160.8	156.8	618.3
IBI error, detected beats (ms)	38.7	24.6	41.3	26.6	17.3 *
HR error (bpm)	1.27	0.90	1.56	0.82	2.49
Peak-timing SD (ms)	49.9	39.3	53.0	40.2	27.5 *
Pulse-to-pulse correlation	0.948	0.975	0.947	0.971	0.967

**Table 10:** Paired participant-level differences between each reference method and the proposed method (27 test participants): median difference [95% bootstrap CI]; *p* value of the Wilcoxon signed-rank test. For error metrics, positive differences indicate a larger error of the reference method; for Se, PPV, and correlation, positive differences indicate a higher value of the reference method. Median differences of 0.00 with significant *p* values indicate that the paired differences were zero in most participants and favored the reference method in the remaining ones.

Metric	Band-Pass − Proposed	Db4 − Proposed	NLMS − Proposed
Se	−0.03 [−0.09, 0.00]; <0.001	0.00 [0.00, 0.00]; 0.031	−0.02 [−0.05, 0.00]; <0.001
PPV	0.00 [0.00, 0.04]; 0.005	0.00 [0.00, 0.00]; 0.27	0.00 [0.00, 0.03]; 0.001
IBI error, missed beats included (ms)	45.6 [3.4, 74.7]; <0.001	14.3 [9.1, 17.5]; <0.001	31.2 [−0.1, 45.9]; 0.004
IBI error, detected beats (ms)	−3.1 [−6.1, −1.1]; 0.005	6.8 [3.8, 10.5]; <0.001	−3.6 [−7.4, −2.3]; <0.001
HR error (bpm)	0.00 [−0.05, 0.14]; 0.98	0.38 [0.22, 0.49]; 0.004	−0.03 [−0.12, 0.01]; 0.093
Peak-timing SD (ms)	−3.0 [−8.2, 0.1]; 0.009	5.0 [3.1, 8.0]; <0.001	−2.1 [−5.5, −1.4]; 0.002
Pulse-to-pulse correlation	0.020 [0.011, 0.031]; <0.001	−0.005 [−0.007, −0.002]; 0.019	0.006 [0.003, 0.012]; 0.002

**Table 11:** Proposed method versus band-pass filtering by motion cluster in the 27 test participants. Values are medians across participants of the per-participant median; *p* values (Wilcoxon signed-rank test) are given in parentheses. Because the test is paired, its direction can differ from that suggested by the two medians (see text for cluster 1).

Metric (Proposed/Band-Pass)	Cluster 1	Cluster 2	Cluster 3
Test windows	32,347	8597	7697
Se	1.00/1.00 (0.001)	0.92/0.82 (<0.001)	0.85/0.70 (<0.001)
IBI error, missed beats included (ms)	89.9/43.0 (0.028)	206.8/253.9 (<0.001)	279.9/388.7 (<0.001)
IBI error, detected beats (ms)	32.4/19.3 (<0.001)	48.7/40.1 (0.43)	85.5/70.4 (0.28)
HR error (bpm)	1.14/0.82 (0.14)	1.55/1.94 (0.034)	3.05/4.38 (0.019)

## Data Availability

The data supporting the reported results are not publicly available because of ethical and privacy restrictions, but they are available from the corresponding author upon reasonable request. The MATLAB implementation and trained model parameters are also available from the corresponding author upon reasonable request, subject to applicable institutional and intellectual-property requirements.
